# ﻿A revision of the Chilean water penny genus *Tychepsephus* Waterhouse, 1876 (Coleoptera, Psephenidae, Eubriinae), with description of a second species and two larval morphotypes, and notes on other Chilean Psephenidae

**DOI:** 10.3897/zookeys.1164.103184

**Published:** 2023-05-26

**Authors:** William D. Shepard, Cheryl B. Barr

**Affiliations:** 1 Essig Museum of Entomology, University of California, 1101 Valley Life Sciences Bldg., Berkeley, CA 94720, USA University of California Berkeley United States of America

**Keywords:** Aquatic beetles, biology, distribution, habitat, life stages, neotropical, sexual dimorphism, South America, synonym

## Abstract

The Chilean water penny genus *Tychepsephus* Waterhouse, 1876 is revised, with descriptions and photographic illustrations of life stages including two larval morphotypes, the pupa of one morphotype, and adults of two species. The pupa of *Tychepsephus* has not been reported previously. *Tychepsephuscekalovici***sp. nov.** is described, and Ectopria (Chilectopria) grandis Pic, 1947, **syn. nov.** is proposed as a new synonym of *Tychepsephusfelix* Waterhouse, 1876, which is redescribed. Taxonomic treatment of the adults of both species includes images of the habitus of males and females, morphological variation, and male and female genitalia. Males and females are sexually dimorphic. Information on the habitat of *Tychepsephus* is provided and illustrated with photographs, and the known geographic distribution of the two species is mapped. The occurrence of *Tychepsephus* in Argentina is reported; therefore, the genus no longer can be considered endemic to Chile. The taxonomic status and geographic distribution in South America of other species of Psephenidae, particularly members of the subfamily Eubriinae, is reviewed.

## ﻿Introduction

Members of the family Psephenidae, commonly called water penny beetles, occur on all continents except Antarctica, and are absent from many islands including New Zealand, Hawaii, and Ireland (Lee et al. 2016). The larvae are found in diverse aquatic habitats and mature over multiple years. The adults are mostly terrestrial in nearby riparian vegetation or debris, do not feed, and are short-lived. Pupae are almost always terrestrial.

The subfamily Eubriinae is cosmopolitan and presently consists of 15 described genera. Larval eubriines, depending on larval morphology, live in a variety of habitats ranging from sluggish seeps to moderately swift streams and small rivers. However, the majority, which are not strongly flattened and streamlined, inhabit slowly flowing water. *Tychepsephus* Waterhouse, 1876, until now a monotypic genus, has been known only from Chile. Larvae have been found on the substrates of very small streams to small rivers with moderate to fast current; adults have been collected from adjacent vegetation.

The taxonomy of *Tychepsephus* has been muddled. Waterhouse (1876) described the genus *Tychepsephus* and the species *T.felix* from “Chili” and placed them in the family Psephenidae. Philippi (1887) misspelled the genus as “*Tychepselaphus*” and placed the genus in the family Pselaphidae. Blackwelder (1944) misspelled the genus as “*Tychepsephenus*” and listed *T.felix* under Psephenidae: Psepheninae in his catalog of neotropical Coleoptera, as did Moroni (1985) in his list of Chilean aquatic beetles. The generic name was misspelled as “*Tychepsephenus*” by many authors, but not all, for more than one hundred years dating from Zaitzev (1910) through Passos et al. (2018).

Elgueta and Arriagada (1989) added two species to the list of Chilean Psephenidae, Ectopria (Chilectopria) grandis Pic, 1947 and *Eubrianaxluteosignatus* Pic, 1947, that had been placed in Dascillidae (Pic 1947). Jerez and Moroni (2006) listed *T.felix* and Ec. (C.) grandis in Psephenidae: Eubriinae, and *Eu.luteosignatus* in Psephenidae: Eubrianacinae, currently accepted subfamily placements. They also cited *Tychepsephus* as a faunal connection of Chile to Australia and New Zealand, and reported the presence of larval *Tychepsephus* specimens in the Museo de Zoologia de la Universidad de Concepción. Zarges et al. (2019) listed three psephenid genera (*Tychepsephus*, *Ectopria* LeConte, 1853 and *Eubrianax* Kiesenwetter, 1874), and noted that the larvae are found in areas with “high slope, with high speeds of currents, low and stable temperatures, and high concentrations of oxygen,” citing *T.felix* as an example. Ashworth and Hoganson (1987) collected adults of *T.felix* in the Central Valley adjacent to Parque Nacional de Puyehue by “trampling water marginal vegetation.”

There has been a question of how closely related *Tychepsephus* is to the Australian eubriine *Sclerocyphon* Blackburn, 1892. In her revision of *Sclerocyphon*, Davis (1986) considered the two genera to be potentially congeneric. Lee et al. (2007) constructed a phylogeny of Psephenidae based on morphological characters of the larvae, pupae, and adults. Their phylogenetic tree placed the two genera as distinct but sister genera near the base of the Eubriinae. In their analysis of the Eubriinae, there was a basal trichotomy with the pair (*Sclerocyphon* + *Tychepsephus*) sister to Eubriinae genus A (since described as *Neoeubria* Shepard & Barr, 2014) and the rest of the Eubriinae genera. When this phylogeny was constructed, the pupae of *Tychepsephus* and Eubriinae genus A (*Neoeubria*) were unknown.

The larva of *Tychepsephus* has also been taxonomically misinterpreted and the generic name incorrectly spelled. Lataste (1897a) published a fairly extensive and accurate description of a “crustacéiforme” larva from near Peñaflor, Chile, that she assigned to the “Pseudo-Névroptère” *Prosopistoma* Latreille, 1833 (now in Ephemeroptera: Prosopistomatidae). However, before that description was published, Lataste discovered that what she described was actually a coleopteran associated with the “Elmides et de Parnides” and issued a correction (Lataste 1897b), but she never associated a specific name with her larva. Later, Artigas (1963) described and illustrated a psephenid larva from the south-central zone of Chile which he believed to be the larva of *T.felix*, although he misspelled the genus as “*Tychepsephenus.*” A key to the genera of neotropical psephenid larvae was published by Passos et al. (2018) in which the name was also misspelled as “*Tychepsephenus.*”

The initial description of larval *Tychepsephus* (Lataste 1897a) as being “crustacéiforme” is similar to the initial description of the larvae of *Psephenusherricki* (DeKay, 1844), the type species of *Psephenus*, which was originally described as an isopod crustacean (Brown 1983: 1). Other aquatic insect larvae which may be mistaken for *Tychepsephus* larvae, and that have been labelled as such, include various cased Trichoptera larvae and the larvae of Diptera (Blephaceridae: *Edwardsinachilota* Edwards, 1929; Psychodidae: *Maruina* Müller, 1895).

In 2005, at a meeting of the Sociedad Chilena de Entomologia, Elgueta and Guerrero presented a talk entitled “Nuestro Conocimiento de Psephenidae (Coleoptera) en Chile” in which they reviewed the taxonomy, morphology, and biology of the family in general, and of the Chilean taxa in particular. The state of knowledge of Chilean species and future directions for research were discussed (Elgueta and Guerrero 2005).

We conducted a survey of the Chilean aquatic Dryopoidea during 2002–2008, and in the process, discovered an undescribed species of Psephenidae. The primary objectives of this paper are to further clarify the taxonomy of *Tychepsephus*, including the proposal of a new synonymy, to describe the new species and redescribe the type species, and to provide new biological, ecological, and geographical information.

## ﻿Materials and methods

### ﻿Institutional acronyms and other abbreviations

**CASC** California Academy of Sciences, San Francisco, California, USA

**CONOCET** CONICET-UNPSJB, Chubut, Argentina

**EMEC** Essig Museum of Entomology, University of California, Berkeley, California, USA

**MNHN** Muséum National d’Histoire Naturelle, Paris, France

**MNNC** Museo Nacional de Historia Natural, Santiago, Chile

**MCZC** Museum of Comparative Zoology, Harvard University, Cambridge, Massachusetts, USA

**NHMUK** The Natural History Museum, London, UK

**UMC** nns Entomological Museum, University of Missouri, Columbia, Missouri, USA

The following abbreviations are used in the text: **morph** = morphotype; **AB** = larval abdominal segment; ***Ec.*** = *Ectopria*; ***Eu.*** = *Eubrianax*; **lin.** = lines (obsolete, small English unit of length, varying from 1/10–1/40 in.); **Pte.** = Puente (Spanish for “bridge”); **WDS** = William D. Shepard.

### ﻿Aquatic sampling

Aquatic sampling of small rivers and streams was conducted during four trips to Chile during the austral summer (December through March) in 2002, 2002–2003, 2007, and 2008. Larvae were collected from the substrate of watercourses by disturbing the gravel and cobbles upstream from an aquatic net. Adult specimens were swept or beaten from adjacent riparian vegetation. Specimens were preserved in vials of ethanol.

### ﻿Study material

In total, 103 adult specimens of *Tychepsephus* were examined during the study and are, or will be, deposited in the various institutions listed above. The larval specimens were not counted, but several hundred were collected. Only one pupa was examined. All larvae and the pupa will be deposited in the EMEC. The types of Ectopria (Chilectopria) grandis and *Eubrianaxluteosignatus* were borrowed from the MNHN for examination. The type of *Tychepsephusfelix* and other specimens of the species at the NHMUK were examined in London, and photographs were later supplied by the museum.

### ﻿Laboratory procedures

An American Optical Spencer stereo microscope fitted with a calibrated ocular grid was used for examination and measurement of specimens, as well as a Leica MZ 125 stereomicroscope fitted with a micrometer. Measurements of total body length represent the length of the pronotum plus the length of the elytra, excluding the head and the variable space between the pronotum and elytra. Measurements of body width include both elytra at their widest point. Specimens with separations between the elytra were not measured for width; therefore, the reported length and width measurements may have different “*n*” numbers. Genitalia from selected male and female specimens were dissected, examined, placed in genitalia vials containing a drop of glycerin and pinned beneath the point-mounted specimens. The terminology used to describe the larvae and pupa follows that of Lee et al. (2007, 2016).

### ﻿Specimen label data

Complete label data, not necessarily verbatim, are reported in the “Material examined” sections. Additional clarifying details not found on the labels are provided within square brackets “ []”. More complete locality data for all samples containing *Tychepsephus* are presented in Appendix 1. Some of the geographic coordinates and elevations, which were taken in the field in 2002 and 2003 using a hand-held GPS unit, were subsequently discovered to be somewhat inaccurate. In the years following our fieldwork, governmental changes were made to some of the Chilean regional designations. Two regions that were split affect some of our label data: Región Ñuble (XVI) was formed from the northern part of Región Bío Bío (VIII), and Región Los Ríos (XIV), from the northern part of Región Los Lagos (X). Our specimen labels and the data in Appendix 1 reflect the situation at the time of collection.

### ﻿Specimen imaging and distribution mapping

Habitus images by the authors were taken using a Visionary Digital BK Plus Lab System fitted with a Canon EOS 7D camera. Images of holotypes were provided by The Natural History Museum, London, UK and the Muséum National d’Histoire Naturelle, Paris, France, as indicated in figure legends. Rachael Diaz-Bastin (California Academy of Sciences) took the genitalic images using a Syncroscopy AutoMontage system. Images were prepared and assembled into plates using Adobe PhotoShop Elements. Additional digital photographs were provided by Nicolás Román (CONICET), Mario Elgueta, Marcelo Guerrero (MNNC) and Robert Sites (UMC).

SimpleMappr, a free internet program (Shorthouse 2010), was used to create the adult distribution map. Geographical coordinates taken in the field were verified with Google Earth Pro for use in low-resolution mapping in the SimpleMappr format. These data were obtained from Google Earth Pro imagery containing the following attribution: “Data SIO, NOAA, U.S. Navy, NGA, GEBCO; Image Landsat / Copernicus; Imagery Date: 12/13/2015.”

## ﻿Taxonomy

### ﻿Subfamily Eubriinae

Taxon classificationAnimaliaColeopteraPsephenidae

Lacordaire, 1857

9E58A9A6-BA81-54D5-94D9-F3D7F405ECAB

#### Type genus.

*Eubria* Latrielle, 1829

#### Diagnosis.

The following characters, in combination distinguishing the Eubriinae from the other four psephenid subfamilies, Afroeubriinae, Eubrianacinae, Psepheninae and Psephenoidinae, are for the most part taken from Lee et al. (2007, 2016). **Adults**: 1) dorsally convex body (flattened in other subfamilies except Afroeubriinae); 2) anterior margin of pronotum truncate or emarginate with an exposed head (Eubrianacinae with pronotum rounded anteriorly and head entirely concealed); 3) maxillary palpus with apex not tapering (i.e., truncate, rounded or bifurcate) (tapering in Afroeubriinae); 4) apex of the mesosternal process truncate or emarginate (Afroeubriinae with process acute; Psephenoidinae with process tapered); and 5) five abdominal ventrites (Psepheninae with seven ventrites in males and six in females). **Larvae**: 1) abdominal paratergites VII not lengthened to reach abdominal segment IX (reaching anterolateral angles of IX in Afroeubriinae and Psepheninae; surrounding IX in Psephenoidinae); 2) ventral external gills absent (present in Eubrianacinae and Psepheninae); and 3) mature larvae metapneustic with a pair of spiracles near bases of abdominal paratergites VIII (Afroeubriinae metapneustic with spiracles at apices of paratergites VIII; Eubrianacinae and Psepheninae amphineustic with exposed ventral gills; Psephenoidinae apneustic).

#### Geographic distribution.

The Eubriinae occur almost worldwide except in Antarctica and on some islands, including New Zealand. The subfamily is represented by 15 genera and 144 species (Lee et al. 2016; Barr and Shepard 2017), with the greatest diversity in Asia. The genera *Dicranopselaphus* Guérin-Méneville, 1861, *Eubria*, *Neoeubria*, and *Tychepsephus* occur in the Neotropics. Of these, only *Neoeubria* and *Tychepsephus* are known from South America, although *Dicranopselaphus* possibly occurs there as well.

#### Habitat and biology.

See Lee et al. (2016) for an overview of the subfamily. See Shepard and Barr (2014) and Barr and Shepard (2017) for habitat descriptions of two neotropical species in the genera *Neoeubria* and *Eubria*, respectively.

#### Remarks.

In Chile, the only known psephenids are eubriines in the genus *Tychepsephus*, and the species currently named *Eubrianaxluteosignatus*. The type of *Eu.luteosignatus* appears to be a eubriine, so it is likely misplaced in *Eubrianax* which is in the subfamily Eubrianacinae. Elgueta and Guerrero (2005) stated that all known Chilean psephenids are eubriines.

### ﻿Genus Tychepsephus

Taxon classificationAnimaliaColeopteraPsephenidae

Waterhouse, 1876

C7BAC35D-48F4-5556-97FA-473DD45371E9

Tychepsephus Waterhouse, 1876: 15.
Ectopria (Chilectopria) Pic, 1947: 3–4, syn. nov.
Tychepselaphus : Philippi, 1887: 665, lapsus calami.
Tychepsephenus : Zaitzev, 1910: 4, lapsus calami.
Tychepsephenus : Blackwelder, 1944: 274, lapsus calami.
Tychepsephenus : Artigas, 1963: 6, lapsus calami.
Tychepsephenus : Moroni, 1985: 173, lapsus calami.
Tychepsephenus : Ashworth & Hoganson, 1987: 879, lapsus calami.
Tychepsephenus : Passos et al., 2018: 598, lapsus calami.

#### Type species.

*Tychepsephusfelix* Waterhouse, 1876, by monotypy.

#### Etymology.

Waterhouse (1876) did not explain the etymology of the genus. However, *Tyche* (Gr.) refers to the goddess of fortune; and the suffix, –*psephus*, from *psephenos* (Gr.), meaning “dark, obscure,” has been used as the stem for multiple psephenid genera.

#### Adult diagnosis.

The following characters used to distinguish *Tychepsephus* from the neotropical eubriine genera *Dicranopselaphus*, *Eubria*, and *Neoeubria* are for the most part taken from Lee et al. (2007, 2016): 1) antenna of the male weakly serrate (pectinate in *Neoeubria*); 2) pronotum with serrate lateral margins (not serrate in *Dicranopselaphus* and *Eubria*); 3) mesoventrite with a median longitudinal sulcus (absent in *Eubria* and some *Dicranopselaphus*); and 4) metaventrite with a transverse suture (vestigial in other eubriines except *Sclerocyphon*). For the original characterization of adults of *Tychepsephus*, see Waterhouse (1876: 15–16).

*Tychepsephus* and the Australian genus *Sclerocyphon* are closely related sister genera which, in the adult stage, do not have many good characters to separate them. One obvious difference is that in *Tychepsephus* the apical margins of abdominal ventrites 2–4 are entire, whereas in *Sclerocyphon* they are serrate. Of course, there is also the geographical difference with each occurring on different continents: *Tychepsephus* in South America and *Sclerocyphon* in Australia.

#### Adult description.

Waterhouse (1876) described the genus *Tychepsephus* in English, unlike the type species description which is in Latin. His description is adequate for identifying the genus. However, since Waterhouse had only a female specimen, he did not know that his species, *T.felix*, is sexually dimorphic, with males having a distinctive patch of setae on the abdominal venter. This character is also present in *T.cekalovici* sp. nov. The dorsal patterning also differs between the sexes of both species.

#### Remark, adult males.

A new morphological note is that males have a sperm pump composed of a heavily muscularized ejaculatory duct which is situated medially, anterior to the aedeagus, bent around itself in an S-shape and coming from the juncture of the paired testes. The sperm pump is as short as or shorter than the aedeagus.

#### Larval diagnosis.

The following characters used to distinguish *Tychepsephus* from the neotropical eubriine genera *Dicranopselaphus*, *Eubria*, and *Neoeubria* are for the most part taken from Lee et al. (2007, 2016): 1) antennomere 1 subequal to antennomere 2 (much shorter in other eubriines except *Sclerocyphon*); 2) maxillary and labial palpi with 4 and 3 palpomeres, respectively (*Dicranopselaphus* and *Eubria* with 3 and 2, respectively); 3) longitudinal medial suture from thorax to abdominal segment VII (not in other eubriines except *Sclerocyphon*).

Larval characters separating *Tychepsephus* from its Australian sister genus *Sclerocyphon* include the following: 1) setae on the posterior margin of the thoracic and abdominal tergites hair-like in *Tychepsephus* and absent in *Sclerocyphon*; 2) paired longitudinal rows of setae or sensillae near the dorsal midline in *Sclerocyphon*, but not in *Tychepsephus*; and 3) gin traps on the abdominal tergites of *Sclerocyphon*, but not on *Tychepsephus*.

#### Larval descriptions.

**Figures 1, 2.**
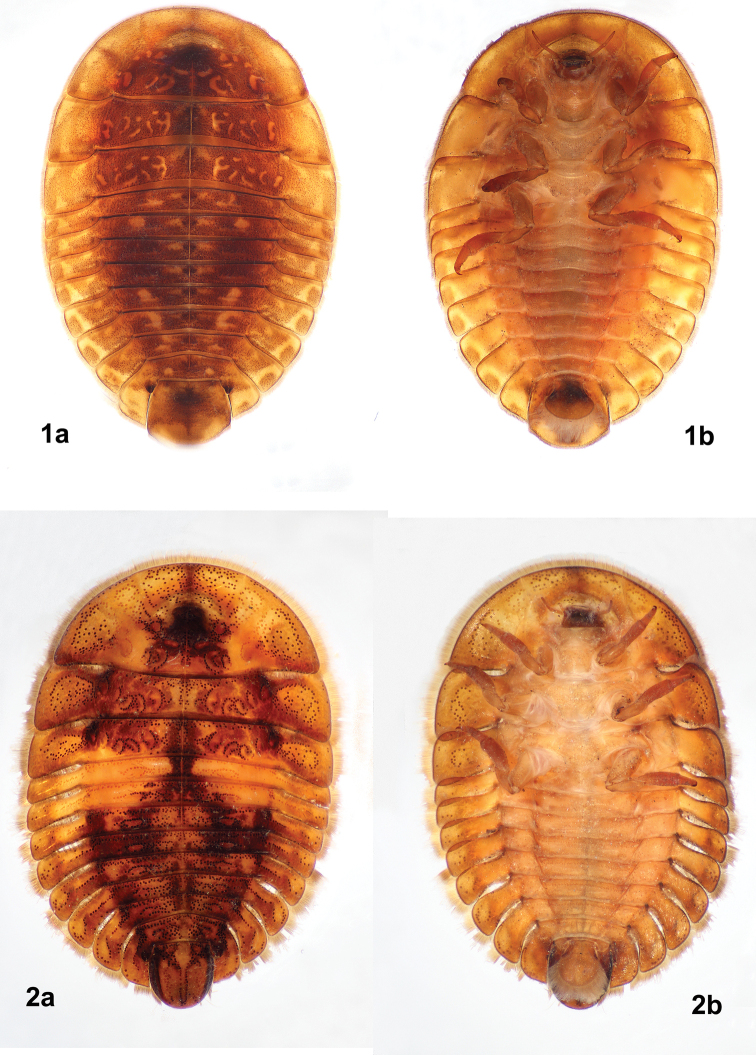
*Tychepsephus* larvae **1**morph 1 **a** dorsal view **b** ventral view **2**morph 2 **a** dorsal view **b** ventral view.

*Tychepsephus* larvae were previously well-described by Lataste (1897a) and Artigas (1963). Our collections revealed the presence of two distinct larval morphotypes (Figs 1, 2) which have not been associated with adults and are therefore unnamed. Artigas (1963) illustrated a larva that corresponds to our larval morph 2, below.

***Tychepsephus* larval morph 1 (Fig. 1).** Body shape elongate-oval; color brown and red-brown with variable yellow patterning; tergites darker than paratergites. Body margined with a long, dense fringe of golden yellow, white-tipped setae. Dorsal surface with very small, scattered cuticular beads. Longitudinal medial suture from middle of pronotum to AB VIII tergite. Mesothoracic tergite to AB VII tergite each with a pair of large, prominent, dark tubercles straddling the medial suture. Abdominal tergite VIII clasping AB IX laterally; AB VIII with a pair of large, spiracular tubercles on posterior margin at bases of paratergites. Abdominal tergite IX subquadrate, flattened, apex rounded. Paratergites generally rectangular, more than twice as wide as long, anterolateral margin curved. Ventral surfaces lacking tubercles and cuticular beads. Abdominal ventrites I–VIII with sternopleural sutures. Operculum as long as wide, widest just anterior to apex; apex broadly rounded.

***Tychepsephus* larval morph 2 (Fig. 2).** Body shape elongate-oval; color brown to red-brown with yellow areas of variable size, shape, and position, often along midline of tergites and base of paratergites. Body margined with a long, dense fringe of yellow-brown, white-tipped setae. Dorsal surface sculptured with shallow, irregularly shaped depressions of varying sizes; cuticle with numerous, round, dark, flat-topped tubercles arranged in curvilinear shapes often encircling the depressions. Longitudinal medial suture from middle of pronotum to AB VII tergite. Tergites without pairs of prominent tubercles at the midline. Abdominal tergites each with an irregular, transverse line of tubercles; paratergites often with a longitudinal line of tubercles near the midline. Abdominal tergite VIII with a pair of large spiracular tubercles on posterior margin at base of paratergites. Abdominal tergite IX subelliptical, convex, sometimes sculptured and laterally angulate. Paratergites I–VIII paddle-shaped, twice as wide as long, narrower at base than apex, anterolateral margin curved. Abdominal ventrites with anterior and posterior transverse lines of small, faint, brown tubercles. Abdominal ventrites I–VIII with sternopleural sutures. Operculum nearly circular from midline to apex.

#### Remarks, larvae.

The most obvious differences between the larval morphs are: 1) the presence on the dorsum of numerous dark tubercles, some in curvilinear patterns (morph 2); 2) the presence of pairs of large, prominent tubercles at the midline (morph 1); 3) the shape of AB IX tergite; and 4) the shape of the operculum. The dorsal morphology of larval morph 2 is quite variable in regard to the number and arrangement of tubercles and the amount of sculpturing. Because of this, it would not be surprising if another undescribed species is discovered with further sampling of the adult habitat.

Some larvae have an abundance of peritrich protozoans attached to the venter in a scattered fashion on both sclerites and membranes.

#### Pupal description

**Figures 3, 4.**
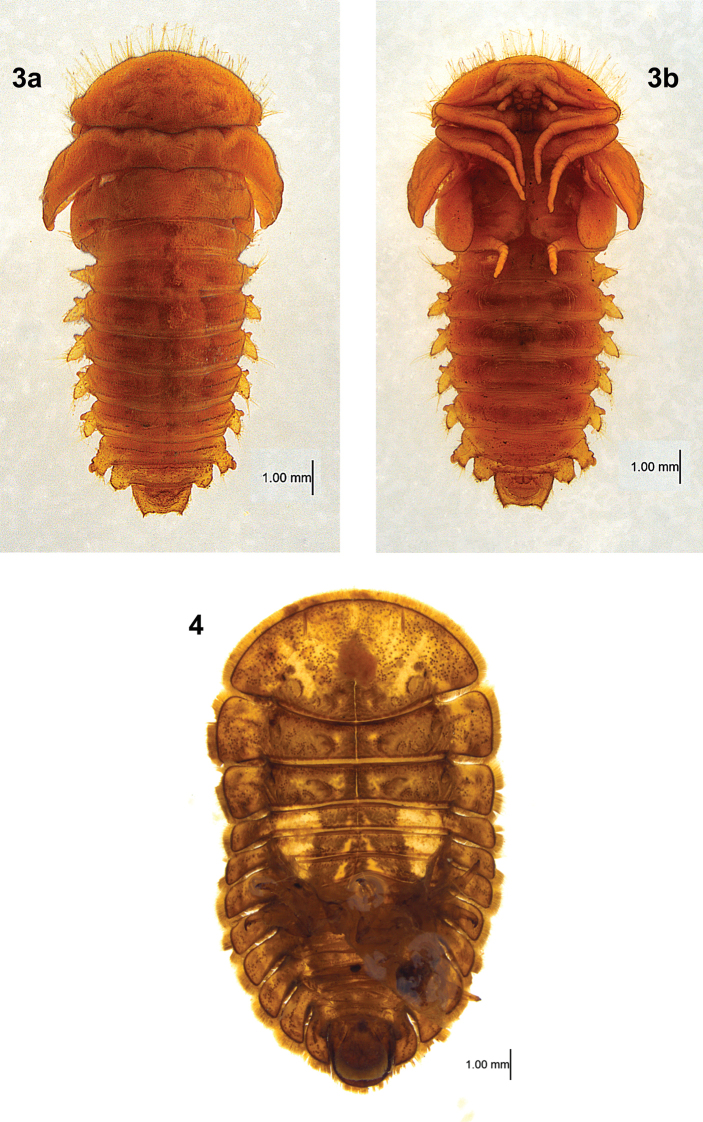
*Tychepsephus* pupa and larval exuvium **3** pupa **a** dorsal view **b** ventral view **4** exuvium of last larval instar, ventral view.

**(pupa of larval morph 2) (Figs 3, 4).** Pupa (Fig. 3) under exuvium of last larval instar (Fig. 4). Exuvium 8.3 mm long; entire dorsum intact; venter with abdominal ventrites anterior to AB II separated from tergum and reflexed, remainder of exuvium intact. Pupa 7.1 mm long, exarate, unsclerotized, color golden yellow. Pronotum projecting anteriorly, covering head; entire margin with very long setae. Elytra and abdominal segments I–IX with long setae on lateral margins. Abdominal tergites I–VIII each with two faint, transverse rows of round tubercles, at anterior 1/3 and near posterior margin, lateral to midline on each side. Abdominal tergite IX with apical margin nearly truncate, each posterolateral angle with a short spine. Paratergites separate; I reduced; II larger, projecting weakly anteriorly; III–VI each longer than II, projecting posteriorly, each with an anterobasal spiracular tubercle; VII similar but with a lateral spiracular tubercle. Ventrally, AB II–VI with sternopleural sutures; II–VIII each with a faint, transverse row of round tubercles near posterior margin.

#### Remarks, pupa.

This rare specimen was provided to WDS by Tomás Čekalović who collected it as a larva at Estero Nonguén in Concepción Province (Región VIII, Bío Bío) on 27 January 1996. He kept it alive for more than nine months until it pupated on 10 November 1996.

In comparison with sister genus *Sclerocyphon*, pupal characters separating the two genera include the following: 1) gin traps in *Sclerocyphon* but absent in *Tychepsephus*; 2) spiracle on abdominal paratergite II reduced in *Tychepsephus* but not in *Sclerocyphon*; 3) spiracles on all paratergites located at the anterior base in *Tychepsephus* but mid-dorsally in *Sclerocyphon*; 4) paratergites in *Sclerocyphon* much longer than in *Tychepsephus*; and 5) apex of abdominal segment IX with a median projection in *Sclerocyphon* but lacking in *Tychepsephus*. The latter two characters probably aid the pupa of *Sclerocyphon* in crawling out from under the larval exuvium (Smith 1981; Davis 1986). The pupa of *Tychepsephus* remains under the larval exuvium until the adult emerges.

##### ﻿*Tychepsephus* geographic and seasonal distribution

In Chile, published localities describe *Tychepsephus* occurring from Peñaflor (Región Metropolitana) in central Chile (Lataste 1897a), south to Chillán, Tomé and Arauco (Región VIII, Bío Bío; Región XVI, Ñuble) (Artigas 1963), and west of Puerto Varas (Región X, Los Lagos) (Ashworth and Hoganson 1987). Data records from the MNNC include adult specimens from the Andes and foothills in Reserva Nacional Altos de Lircay (Región VII, del Maule), Cordillera Chillán (Región XVI, Ñuble), Parque Nacional Conguillío (Región IX, Araucanía) and Parque Nacional Vicente Pérez Rosales (Región X, Los Lagos), as well as specimens from near the Pacific Coast in Quirihue (Región XVI, Ñuble) and Valdivia (Región XIV, Los Ríos) (M. Elgueta, in litt.). See Appendix 2 for detailed locality information regarding the above records. Collections by the authors of adult and larval *Tychepsephus*, from regions VIII (Bío Bío) through XI (Aysén), plus regions XIV (Los Ríos) and XVI (Ñuble), are listed in Appendix 1.

**Figures 5, 6.**
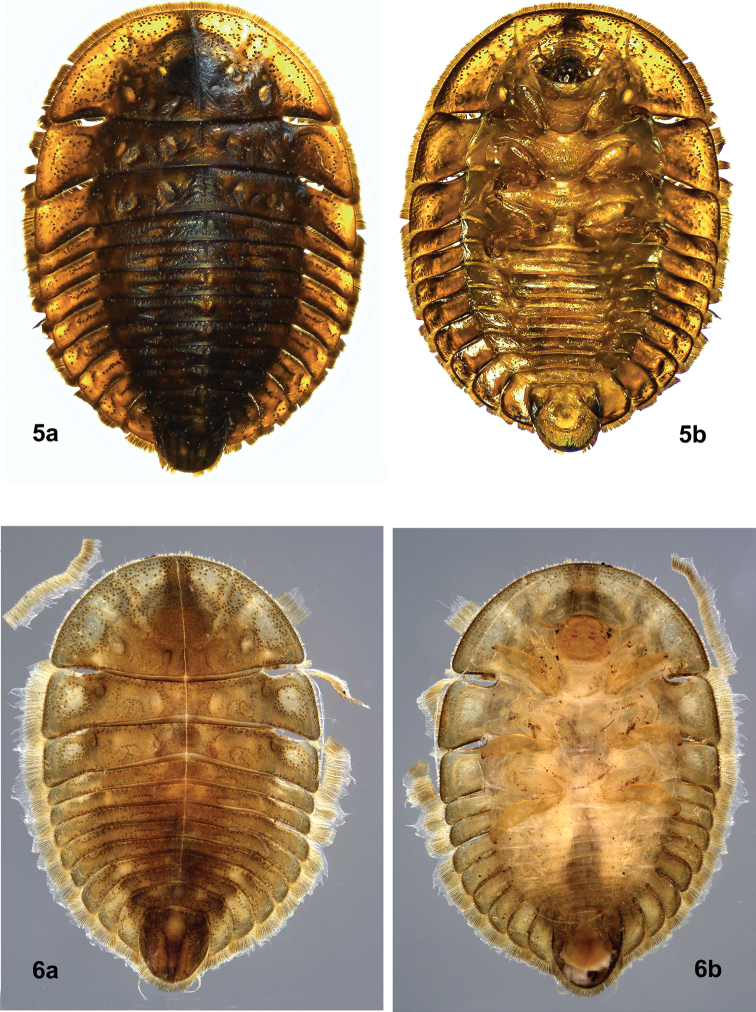
Non-Chilean larvae **5***Tychepsephus* larva from Argentina **a** dorsal view **b** ventral view. Images provided by Nicolás Román (CONICET) **6** larva from French Guiana, possibly *Tychepsephus***a** dorsal view **b** ventral view. Images provided by Robert Sites (UMC).

Larval records from Provincia del Neuquén, Argentina, indicate that *Tychepsephus* also occurs on the east front of the Andes. Wais (1990, 1995) reported T*ychepsephus* (listed as *Chilectopriagrandis*) from the Rio Meliquina in the Rio Negro Basin north of San Carlos de Bariloche. Nicolás Román (N. Román, in litt.) has more recently reported finding a larva (morph 2) (Fig. 5) near San Martin de los Andes. More surprisingly, in the EMEC there is a larval eubriine from French Guiana (Fig. 6) that greatly resembles the larva of *Tychepsephus*. If this is actually a *Tychepsephus* larva, the geographic distribution of the genus would be significantly expanded (see Discussion).

**Figure 7.**
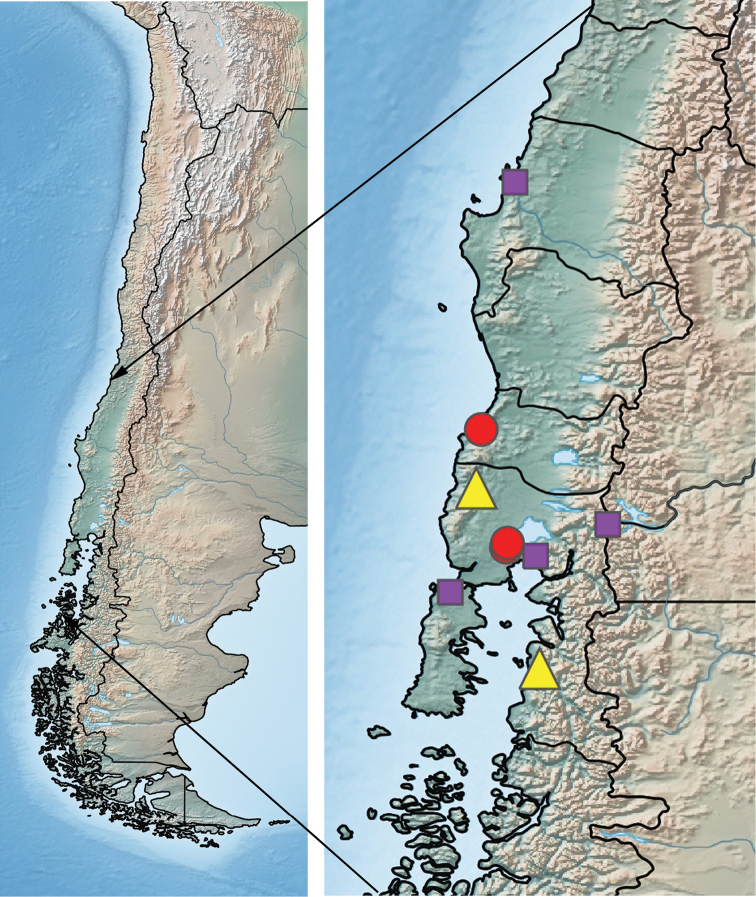
Map of Chile with enlarged section showing the geographic distribution of species-verified adult *Tychepsephus* specimens: *T.cekalovici*, yellow triangles; *T.felix*, violet squares; both species, red circles.

A summation of the locality data for adults and larvae reveals a geographic distribution of *Tychepsephus* in the middle third of Chile, including both the Andes and the Coast Range, and on the eastern front of the central Andes in Neuquén Province, Argentina (Appendices 1, 2). The distribution map (Fig. 7) represents species-verified adult records only.

The adults examined for this study were collected from November through January, during the austral summer. Larvae are present year-round. Tomás Čekalović collected larvae in January and August, and Fierro et al. (2012) reported collecting larval *T.felix* in all seasons except winter.

##### ﻿*Tychepsephus* habitat

We have collected larvae and/or adults of *Tychepsephus* across an elevational range of 15–1685 m at streams and rivers in Chile. In general, these watercourses were small to medium-sized and rather shallow, with moderate current, and with clear or often brown, tannin-stained water. This is in contrast to Zarges et al. (2019) who reported them from areas with “high slope and high water currents.” Label data from specimens collected by Tomás Čekalović show larvae living in temperatures of 10–14 °C. Surprisingly, some them were found in humus under *Chusqueaquila* [Kunth (Poaceae) bamboo], 2–3 m from a river.

**Figures 8–11.**
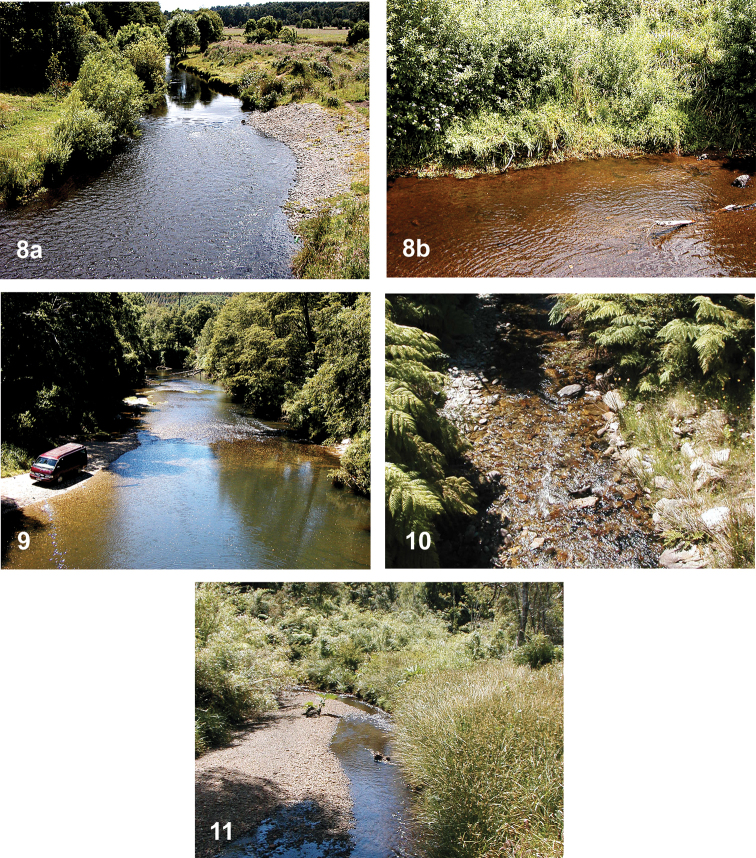
*Tychepsephus* habitat **8** Río Colegual, *T.cekalovici* type locality **a** stream with riffle-pool morphology flowing through a mixture of forest and pasture land **b** streamside shrubby vegetation, adult habitat **9** Río Contaco **10** stream at Puente El Avion **11** tributary Río Blanco.

Examples of the small to medium-sized streams and small, shallow rivers in which adult *Tychepsephus* have been collected by the authors, described below, include Río Colegual, Río Contaco, Río Oroco, and an unnamed tributary of the Río Blanco (Figs 8, 9, 11).

**Río Colegual** (Fig. 8), located west of Puerto Varas at an elevation of ~ 200 m, is a medium-sized stream with moderate to slow flow over cobble and gravel coated with brown algae. Pools with laminar water flow are interrupted by areas of shallow riffles. Riparian vegetation overhangs some of the banks. At the time of sampling, the water was cool, clear, and brown-stained (Fig. 8b). The watercourse is situated in mostly flat terrain bordered by cleared fields (Fig. 8a) and is partly shaded at the collection site near the bridge. Adults of two species were readily collected by sweeping and beating the riparian vegetation, which consisted of willows, streamside grasses and forbs. Blacklight sampling yielded no specimens. Many larvae were present in the substrate of the riffles, including both morphs.

**Río Contaco (= Río Tranallaquín)** (Fig. 9), located west of Osorno at an elevation of ~ 160 m, is a medium-sized stream with clear water, moderate flow, and a substrate of sand, gravel and rubble with submerged mosses. Larvae were very abundant in the substrate of the riffles, as they also were at a small tributary of the Río Contaco at Puente El Avion (Fig. 10).

**Río Oroco** at Puente Hondo, located east of Puerto Montt at an elevation of ~ 30 m, is small and shallow, with cool, brown-stained water and a sand and cobble substrate with aquatic moss.

**An unnamed tributary of Río Blanco** (Fig. 11), located between Caleta Gonzalo and Caleta Santa Bárbara (north of Chaitén) at an elevation of ~ 160 m, yielded one adult and a small number of larvae. The stream is small, clear, and very cold with moderate current, shallow riffles, and knee-deep pools. It has a substrate of cobbles and sandy gravel with some aquatic moss present.

### ﻿ Tychepsephus felix

Taxon classificationAnimaliaColeopteraPsephenidae

Waterhouse, 1876

18DD8E0E-1203-52A1-904B-DA2807D155CD

Figs 7 , 8 , 12 , 13, 14 , 15, 16 , 17

Tychepsephus felix Waterhouse, 1876: 16 (original description). Philippi (1887: 665, catalog); Blackwelder (1944: 274, catalog); Artigas (1963: 8, larval description); Moroni (1985: 173, taxonomy, distribution); Ashworth and Hoganson (1987: 879, habitat, distribution); Elgueta and Arriagada (1989: 16, literature review); Jerez and Moroni (2006: 76, taxonomy, checklist); Lee et al. (2007: 527, phylogenetics), Zarges et al. (2019: 16, habitat).
Ectopria (Chilectopria) grandis Pic, 1947: 4, syn. nov.

#### Type locality.

The type locality was listed as only “Chili” on both the type specimen and in the species description. The female holotype specimen is housed in the NHMUK.

#### Type material.

**Figure 12.**
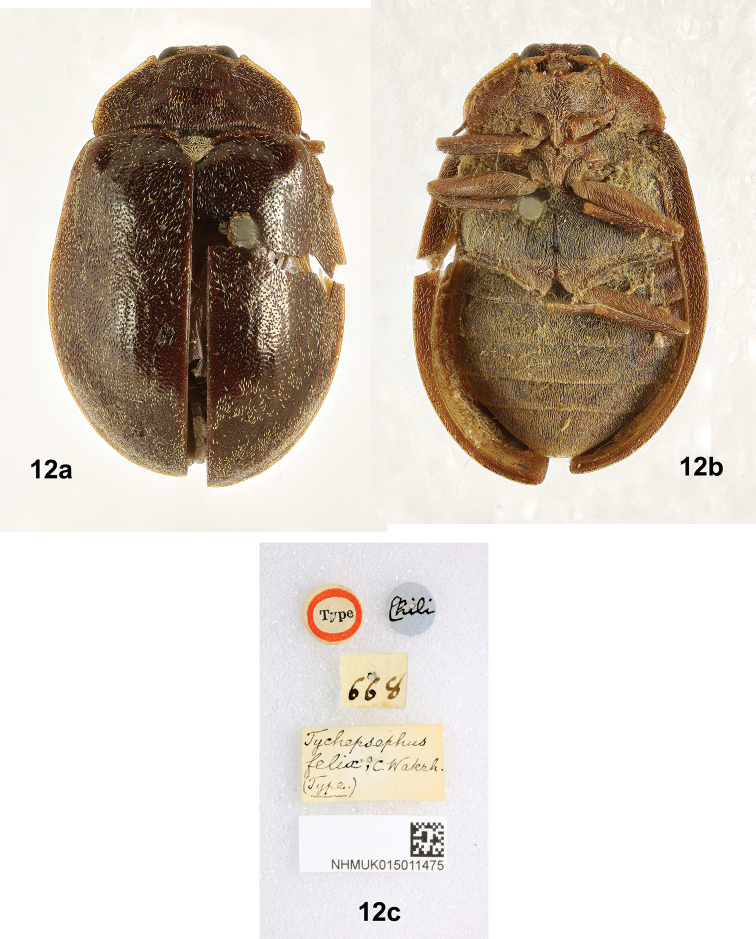
*Tychepsephusfelix*, female type specimen **a** dorsal view **b** ventral view **c** specimen data labels. Images provided by Keita Matsumoto (NHMUK).

*Tychepsephusfelix*, ***Holotype*** female, pinned. Chile: “Type [white, circular label with red border] // Chili [blue, circular label // 668 // Tychepsephusfelix, C. Waterh. (Type.) // NHMUK015011475” (Fig. 12). Deposited in the NHMUK. Ectopria (Chilectopria) grandis syn. nov., ***Holotype*** female, pinned. “Chili [green label] // Dascillide ? [pale brown label] // type [pale brown label] // Museum Paris / Coll. M.Pic [blue-green label] // Chilectopria / s.g. grandis / n sp [pale brown label] // Chilectopria / grandis Pic // TYPE [red label] // HOLOTYPE [red label] // Tychepsephus / felix ♀ / W D Shepard // MNHN, Paris / EC17127” (Fig. 17). Deposited in the MNHN.

#### Other material examined.

Non-types (33). Chile: Region X, 3 km W of Nueva Braunau, Rio Colegual, 30 XII 2002 (WDS-A-1502) [on reverse], William D. Shepard, leg. (EMEC, 5, 2♂ 3♀); Chile: Region X, 9 km E Loncotoro, Pte. Colegual 2, 650' [198 m], 8 I 2003 (WDS-A-1519) [on reverse], William D. Shepard, leg. (11; EMEC, 7, 3♂ 4♀; MNNC, 4, 2♂ 2♀); Chile: Región X Lagos, Río Colegual 8 rd. km W Llanquehue, elev.700' [213 m], 41°16.51'S, 73°06.52'W, 8 Jan. 2003, C. B. Barr, sweeping willows and other riparian vegetation (EMEC, 5, 4♂ 1♀); Chile: Region X, 8 km SW Correntoso, Pte. Hondo [Río Oroco], 420' [128 m], 31 XII 2002 (WDS-A-1504) [on reverse], William D. Shepard, leg. (EMEC, 1♀); Chile: Corral, Dec. 1905, R. Thaxter, MCZ (MCZC, 1♂); Chile: F. C. Bowditch Coll., “Bradytoma”, MCZ (MCZC, 1♂); Chile: Concepción Pr, Estero Nonguen, 11 Noviembre 1996, Tomas Cekálovic (EMEC, 1♀); Chile: Chili, Germain, Sharp Coll. 1905-313, NHMUK015011806 (NHMUK, 1); Chile: Sharp Coll. 1905-313, Tycepsephus [sic] felix, C.O. Waterh., Chili – Germain, NHMUK015011809 (NHMUK, 1); Chile: Puerto Varas, 16.xii.1926, S. Chile: Llanquihue prov., F. & M. Edwards, B.M.1927-63, NHMUK015011807 (NHMUK, 1); Chile: Casa Pangue, 4–10.xii.1926, S. Chile: Llanquihue prov., F. & M. Edwards, B.M.1927-63, Tychepsephus felix Waterh., M.I. Russell det. 1973, NHMUK015011808 (NHMUK, 1); Chile: Ancud. 17–19.xii.1926, S. Chile: Chiloe I., F. & M. Edwards, B.M.1927-63, NHMUK015011810 (NHMUK, 1); Chile: as above, NHMUK015011811 (NHMUK, 1); Chile: Ancud, 19.xii.1926, S. Chile: Chiloe I., F. & M. Edwards, B.M.1927-63, NHMUK015011812 (NHMUK, 1); as above, Tychepsephusfelix Waterh., M.I. Russell det. 1973, NHMUK015011813 (NHMUK, 1).

#### Differential diagnosis.

**Figures 13, 14.**
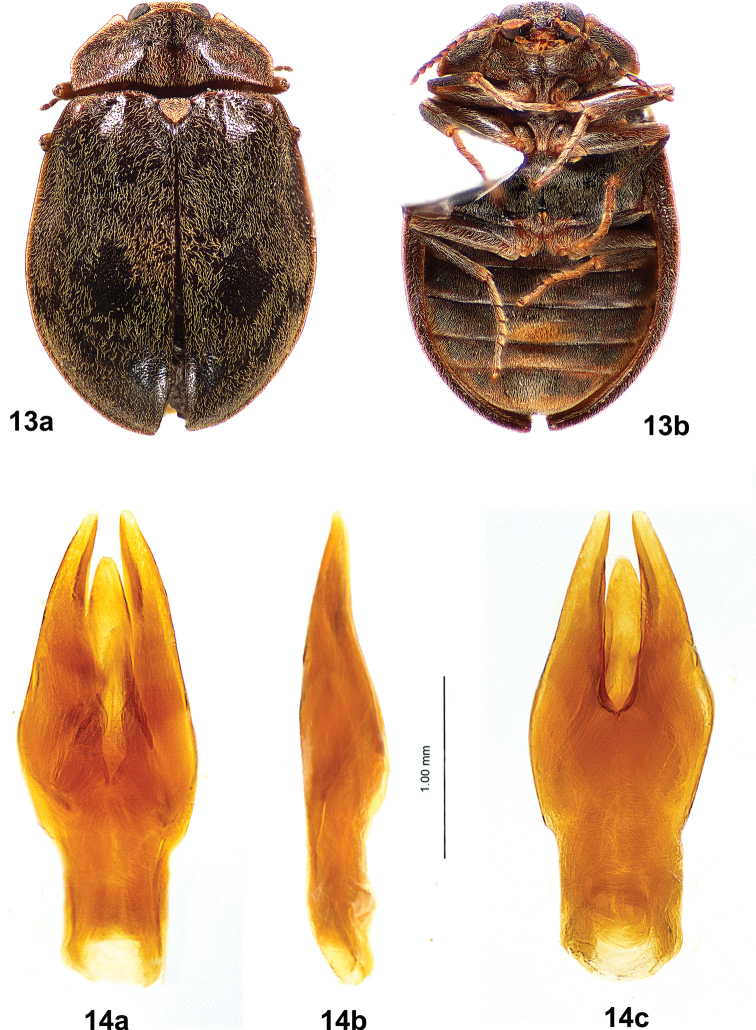
*Tychepsephusfelix*, male **13** habitus **a** dorsal view **b** ventral view; length 5.0 mm **14** aedeagus **a** dorsal view **b** lateral view **c** ventral view.

Males of *T.felix* (Figs 13, 14) are much larger (4.6–5.2 mm long) than those of *T.cekalovici* sp. nov. (3.3–3.9 mm long); the pronotal cuticle is dark brown to black with pale lateral margins, with no yellow markings on disc; the elytral cuticle is dark brown or red-brown, with setal patterning only; the depressed frontal area between the eyes does not have an inverted Y-shaped sulcus; and abdominal ventrite 3 has a median, golden yellow setal patch that is not distinctly raised and does not extend the entire length of the ventrite.

In contrast, males of *T.cekalovici* sp. nov. (Figs 18–20) are considerably smaller than males of *T.felix*; the pronotal cuticle is dark brown to black with pale lateral and basal margins, and a mediobasal yellow spot anterior and adjacent to the scutellar shield; the elytral cuticle is usually yellow-brown with dark markings in a zig-zag pattern, but may be mostly plain, without patterning; the depression between the eyes has a narrow, inverted Y-shaped sulcus; and abdominal ventrite 3 has a prominent, raised, golden yellow setal patch extending the full length of the ventrite.

The aedeagi (Figs 14, 19) of the two species are clearly different. In *T.felix* (Fig. 14), the parameres have straight lateral margins and only slightly curved medial margins, and the apices are narrow. In *T.cekalovici* sp. nov. (Fig. 19) the parameres have curved lateral and medial margins, and the apices are broad.

**Figures 15, 16.**
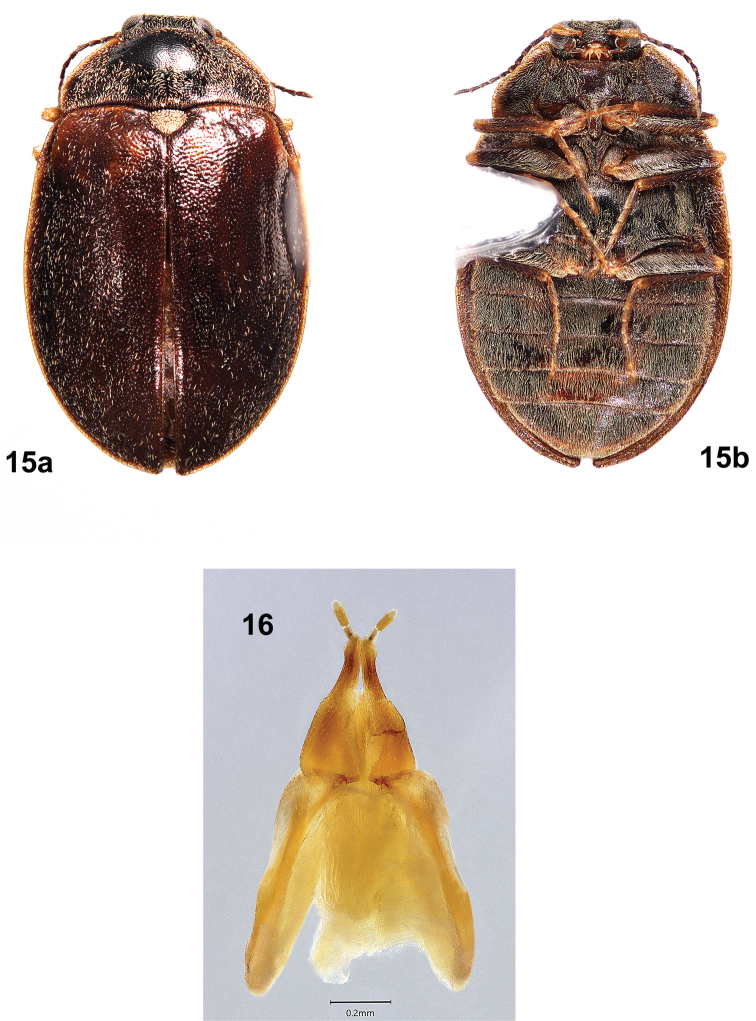
*Tychepsephusfelix*, female **15** habitus **a** dorsal view **b** ventral view; length 4.2 mm **16** ovipositor, dorsal view.

Females of *T.felix* (Figs 15, 16) are much larger (4.3–5.7 mm long) than those of *T.cekalovici* (3.3–3.9 mm long); the elytral cuticle is brown, without yellow patterning except for variable, slightly paler areas near the base; and the pronotal disc has no yellow markings. *Tychepsephuscekalovici* sp. nov. females (Figs 21–23) usually have elytra with transverse yellow bands in a zig-zag pattern, but those without may be distinguished by a mediobasal yellow spot on the pronotum anterior to the scutellar shield. The ovipositors are similar.

#### Original type description, female

**(Fig. 12).**Waterhouse (1876) described the species in Latin, as was the custom at the time. An English translation follows:

Ovate, convex, glossy, dark pitch black, bronzy; fine, short, grey pubescence. Head yellow, rather wide, narrow between antennae, eyes prominent, antennal bases yellow. Thorax slightly convex, densely finely punctate, length twice width, suddenly narrowed anteriorly, frontal margin slightly lobed in middle, both sides sinuate, anterior angles somewhat prominent, sides slightly rounded behind middle, posterior angles almost right-angled, edges narrowly yellow. Scutellum yellow, apex acute. Elytral bases slightly wider than thorax, enlarged posteriorly, arched at apex, narrowed, convex, more clearly finely punctured, dorsum depressed; humeri obtuse, with edges narrowly yellow. Venter with densely grey pubescence, tarsi pitch black-yellow. Length 2.75 lin., width 2 lin.

Waterhouse (1876) added two sentences, in English: “The thorax is at the base nearly straight next to the scutellum, but is broadly sinuate on each side, so that at first sight it appears only bisinuate. Epipleural fold of the elytra is broad at the base, gradually narrowing to the apex, channeled posteriorly.”

#### Redescription based on new material.

**Male** (Figs 13, 14)**. *Body***: covered with short black setae and thick, moderately long yellow setae; yellow setae forming patterns on elytra. Cuticle with closely spaced punctures, punctures finer ventrally. Pronotum black with yellow margins. Elytra dark brown or red-brown with yellow lateral margins. Length 4.6–5.2 mm (*n* = 6), width 2.8–3.5 mm (*n* = 5). ***Head***: covered with moderately long, yellow setae. Vertex between eyes wider than diameter of an eye. Frons deflexed at 90° angle from vertex, with a contiguous pair of broad, moderately deep depressions between eyes. Frontoclypeal suture absent. Clypeus trapezoidal, longer than wide, widest apically; clypeus noticeably raised; anterolateral angles curved beneath antennal bases. Maxillary palpus with four palpomeres; palpomeres 1–3 yellow to yellow-brown; 4 yellow-brown to dark brown, obliquely hatchet-shaped, weakly curved at apex. Labial palpus with three palpomeres; palpomeres 1–2 yellow; 3 yellow-brown to dark brown, with apex truncate to weakly curved; glossae and paraglossae split, apically acicular. Antenna weakly serrate, with 11 antennomeres; 1 longest, cylindrical, yellow; 2 shortest, yellow-brown; 3–11 dark brown, each widest apically. Antennal base encircled by raised margin. Eye large, bulbous, finely faceted. ***Pronotum***: twice as wide as long, widest just anterior to base; anterior margin convex between anterior angles; anterior angles obtuse, broadly rounded, projecting anteriorly, clasping eyes; lateral margins finely sculptured with shallow notches, narrowly explanate, straight from anterior angles to basal 1/3 then curved to posterior angles; posterior angles square; posterior margin crenulate, nearly straight between posterior angles almost to scutellar shield, then curved posteriorly, straight adjacent to scutellar shield; disc depressed near anterior angles, convex at middle, flattened basomedially. ***Scutellar shield***: pentagonal, flat, depressed between elytra; densely covered by moderately long, yellow setae; anterior margin crenulate; apex acute. ***Elytra***: conjointly longer than wide, widest at ~ 1/3 the distance from apices, wider than pronotum; each with anterior margin crenulate; lateral margin smooth, narrowly explanate; humerus prominent with adjacent medial depression; area adjacent to lateral margin in anterior 1/3 with a wide, moderately deep depression; disc much more convex at posterior 1/2. Setae largely absent from oval area posterior to middle and round area at apical 1/4 near suture; setae sparse near base. Epipleuron widest below humerus, narrowed posteriorly; channeled adjacent to abdomen, channel becoming deeper towards apex. ***Metathoracic wings***: macropterous. ***Prosternum***: wider than long; cuticle brown, densely setose; anterior margin projecting to cover mouthparts with a short chin piece; prosternal process long, narrow, extending well past procoxae to anterior third of mesosternum, elevated above rest of prosternum; disc with shallow, longitudinal, median ridge. ***Mesoventrite***: brown; wider than long, extending between mesocoxae; mesoventral cavity deep to receive prosternal process. ***Metaventrite***: wider than long, cuticle black; disc convex lateral to discrimen; deep fossa at junction of discrimen and metakatepisternal suture; posterior margin weakly sinuate anterior to metacoxae. ***Legs***: completely covered with dense, yellow setae; similar, except procoxa and mesocoxa globular, metacoxa widely transverse; femur and tibia usually darker than tarsus; tibia and tarsus long and narrow, tarsus longer than tibia; tarsus densely covered with pale setae; claws long, slender. ***Abdomen***: brown, densely setose; with five weakly convex ventrites; ventrites 1–4 short, ventrite 5 longest; ventrites 1–3 with lateral margins notched medially; ventrite 3 with a dense patch of coarse, golden yellow setae covering the posteromedial 1/2–2/3 of the disc. ***Aedeagus***: (Fig. 14) more than twice as long as wide; phallobase vase-shaped, narrow at base, wide at apex; parameres stout, much longer than penis, lateral margins straight at basal 2/3 then gradually curved inward at apical 1/3, medial margins only slightly curved, parameres each narrowed toward a rounded apex; penis tapered, apex broadly rounded, basally with two apophyses. **Female** (Figs 15, 16) similar to male except: frons with an inverted Y-shaped suture between eyes; setation of dorsum less dense except for scutellar shield; cuticle of humeri and elytral bases sometimes paler than disc; tarsi less densely setose; abdominal ventrite 3 without a dense patch of yellow setae. ***Ovipositor***: (Fig. 16) with baculus nearly twice as long as gonocoxite; baculus almost twice as long as wide, strap-like, wider apically; each proximal gonocoxite triangular, distal gonocoxites separate basally then converging to meet apically, apices obliquely truncate; each gonostylus long and narrow, half as long as distal gonocoxite.

#### Variation.

The sizes of males and females overlap: males, 4.6–5.2 mm long (*n* = 6), 2.8–3.5 mm wide (*n* = 5); females 4.3–5.7 mm long (*n* = 10), 2.9–3.7 wide (*n* = 5); but the largest specimens are female. Most males (Fig. 13a) have an elytral pattern with moderately dense setae surrounding mostly glabrous areas; most females (Fig. 15a) have no pattern and are more sparsely setose. Females (Fig. 15b) lack the dense patch of yellow setae on abdominal ventrite 3 that is present in males (Fig. 13b).

#### Etymology.

Waterhouse (1876) did not explain the trivial name. However, *felix* (L.) means happy or prosperous.

#### Geographic distribution.

*Tychepsephusfelix* is known only from Chile. Adults have been collected mainly in Región X (Los Lagos), but also in regions VIII (Bío Bío), IX (Araucanía), and XIV (Los Ríos) (M. Elgueta, in litt.), in both the Andean and the coastal mountain areas (Fig. 7). The greatest number of adults collected by the authors was at Río Colegual west of Puerto Varas (Fig. 8).

#### Habitat.

*Tychepsephusfelix* adults were found in habitats as described for the genus. Specimens were collected by sweeping marginal vegetation along streams during the austral summer (Fig. 8b).

#### Associated dryopoid taxa.

Elmidae: Larainae: *Hydoraannectans* Spangler & Brown, 1981, *H.lenta* Spangler & Brown, 1981; Elminae: *Austrolimnius* Carter & Zeck, 1929, *Luchoelmis* Spangler & Staines, 2004, *Neoelmississicollis* (Germain, 1892). Both *T.felix* and *T.cekalovici* sp. nov. occurred at Río Colegual.

#### Type remarks.

The species was described by Waterhouse (1876) from a single female type (Fig. 12) which is in the NHMUK along with eight non-type specimens subsequently acquired. The type was originally pinned, breaking the right elytron just posterior to the pin. Subsequently, the specimen was removed from the pin and card-mounted. Missing parts include the following: left antenna without antennomeres 3–11; left foreleg; right hind leg; tarsi on all legs except left hind leg; and tarsomere 5 on the left hind leg. One leg, without the tarsus, is in a gelatin capsule pinned below the carded specimen.

**Figure 17.**
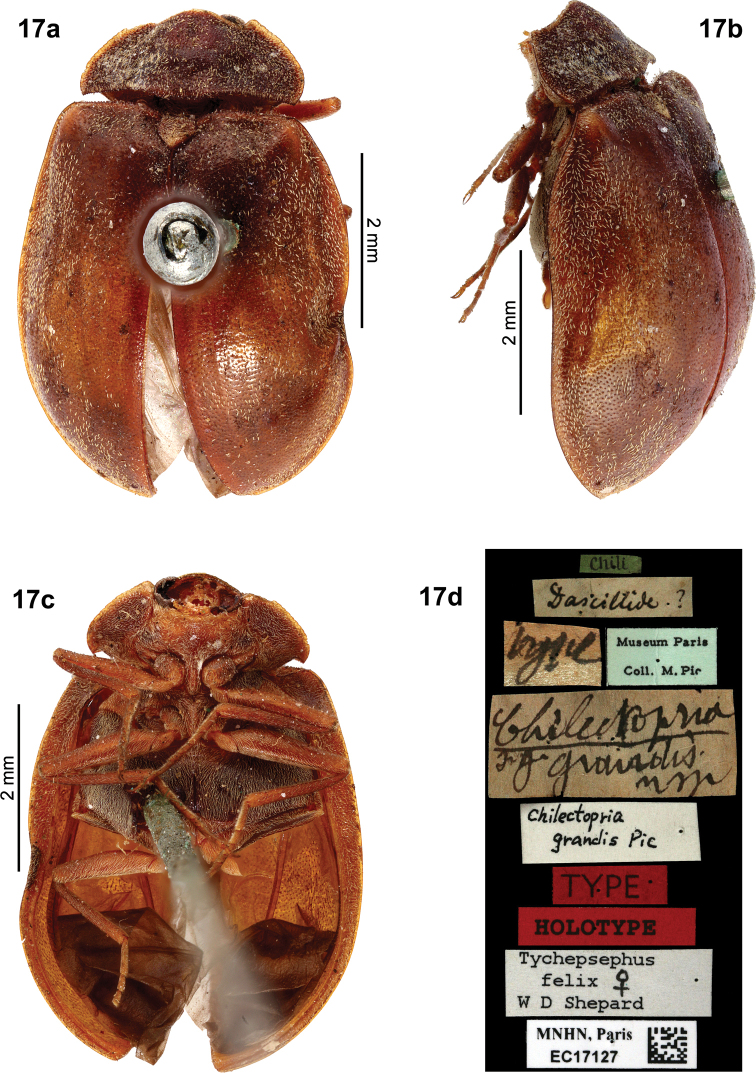
*Chilectopriagrandis*, female type specimen **a** dorsal view **b** lateral view **c** ventral view **d** specimen data labels. Images provided by Christophe Rivier (MNHN).

The female type of Ectopria (Chilectopria) grandis Pic, 1947 (Fig. 17), housed at the MNHN, was examined and found to be synonymous with *Tychepsephusfelix* Waterhouse, 1876. Pic described *Chilectopria* as a subgenus of *Ectopria*: “*Chilectopria* s. g. de *Ectopria.*” Subsequently, *Chilectopria* sometimes has been cited incorrectly as a genus, rather than a subgenus, likely because the heading and description occur on different pages (Pic 1947: 3–4). Contributing to this error, the type specimen also lacks “*Ectopria*” on the identification label. The genus *Ectopria* is a Holarctic element.

### ﻿ Tychepsephus cekalovici sp. nov.

Taxon classificationAnimaliaColeopteraPsephenidae

3726D024-F3A9-540E-B1E8-3EAA875E212C

https://zoobank.org/BEB4F7FE-9984-419A-9D9C-1A08A5255B1E

Figs 7 , 8–9 , 11 , 18–19 , 20 , 21–23

#### Type locality.

Chile: Región X (Los Lagos), 3.5 rd. km W of Nueva Braunau, Puente Colegual, Río Colegual, -41.3264°, -73.1225°, 158 m, sweeping riparian vegetation, 8 January 2003, William D. Shepard leg. (Fig. 8).

#### Type material.

***Holotype*** male, pinned. “CHILE: Region X / 9 km E Loncotoro / 8 I 2003 650' / Pte Colegual 2 [Río Colegual] (WDS-A-1519) [on reverse] // William D. Shepard, leg. // [genitalia in vial below specimen] // HOLOTYPE / Tychepsephus / cekalovici / Shepard & Barr [red handwritten label]”. Deposited in the MNNC.

#### Other material examined.

Paratypes (67). Chile: Region X, 3 km W Nueva Braunau, Rio Colegual, 30 XII 2002 (WDS-A-1502) [on reverse], William D. Shepard, leg. (9; EMEC, 6, 5♂ 1♀; MNHN, 1♂; MNNC, 1♂; NHMUK, 1♂); Chile: Región X Lagos, Río Colegual 3.5 rd. km W Nueva Braunau, elev.160' [49 m], 41°19.58'S, 73°07.35'W, 30 Dec. 2002, C. B. Barr, sweeping willows and other riparian vegetation (11; EMEC, 9, 8♂, 1♀; MNNC, 1♂; NHMUK, 1♂); data as above, 31 Dec. 2002 (EMEC, 5♂); data as above, 8 Jan. 2003 (14; EMEC, 10, 8♂ 2♀; MNHN, 2♂; MNNC, 1♂; NHMUK, 1♀); Chile: Region X, 9 km E Loncotoro, Pte. Colegual 2, 650' [198 m], 8 I 2003 (WDS-A-1519) [on reverse], William D. Shepard, leg. (10; EMEC, 6, 4♂ 2♀; MNHN, 1♀; MNNC, 2, 1♂ 1♀; NHMUK, 1♂); Chile: Región X Lagos, Río Colegual, 8 rd. km W Llanquehue, elev.700' [213 m], 41°16.51'S, 73°06.52'W, 8 Jan. 2003, C. B. Barr, sweeping willows & other riparian vegetation (10; EMEC, 9, 6♂ 3♀; MNNC, 1♀); Chile: 8 mi W of Puerto Varas, 1-16-51, Ross and Michelbacher, CAS (CASC, 1); Chile: Region X, 20 km N Chaitén, unnamed stream [trib. Río Blanco], 520' [158 m], 3 I 2003 (WDS-A-1511) [on reverse], William D. Shepard, leg. (EMEC, 1♂); Chile: Region X, Rio Contaco, 520' [158 m], 9 I 2003 (WDS-A-1521) [on reverse], William D. Shepard, leg. (EMEC, 3, 1♂ 2♀); Chile: Corral, Dec 1905, R. Thaxter, MCZ (MCZC, 3).

#### Differential diagnosis.

**Figures 18, 19.**
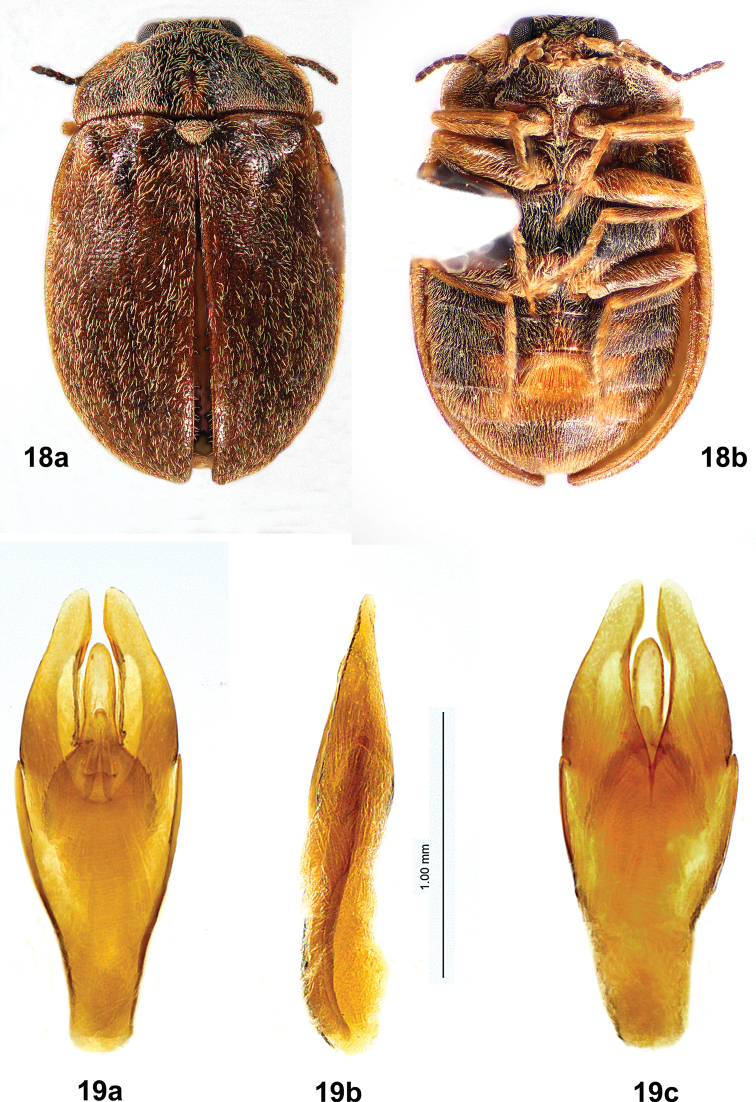
*Tychepsephuscekalovici* sp. nov., male **18** habitus **a** dorsal view **b** ventral view; length 3.2 mm **19** aedeagus **a** dorsal view **b** lateral view **c** ventral view.

**Figure 20.**
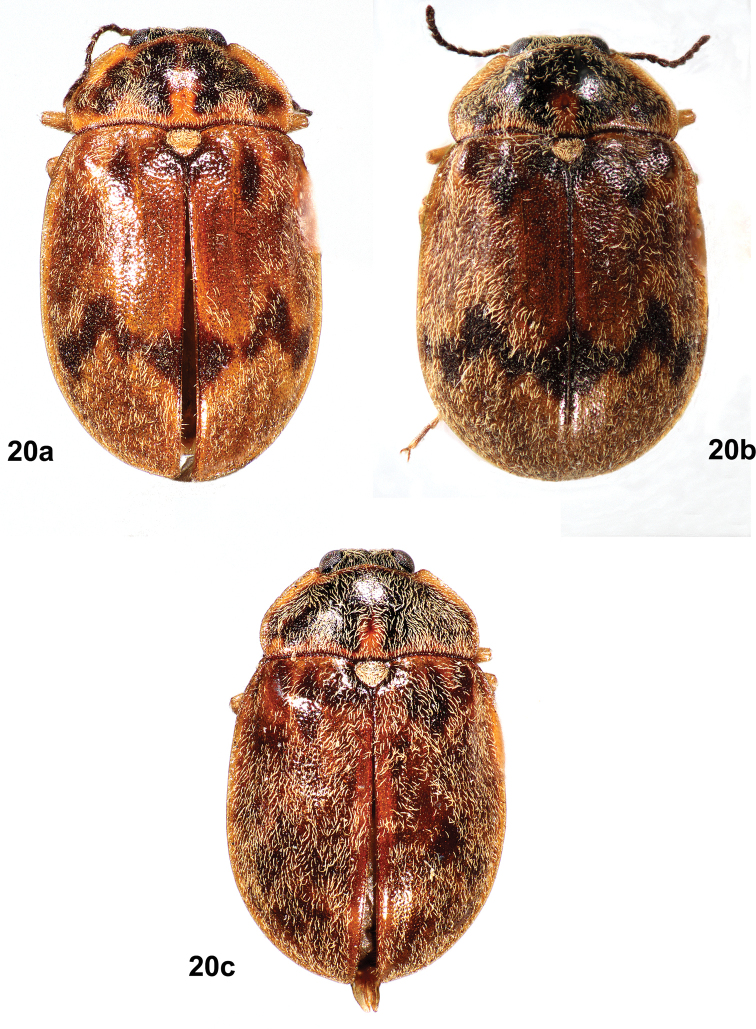
*Tychepsephuscekalovici* sp. nov., male dorsal habitus showing color pattern variation **a** length 3.3 mm **b** length 3.5 mm **c** length 3.5 mm.

Males of *T.cekalovici* sp. nov. (Figs 18–20) are considerably smaller (3.3–3.9 mm long) than males of *T.felix* (4.6–5.2 mm long); the pronotal cuticle is dark brown to black with pale lateral and basal margins, and an elongate yellow spot anterior and adjacent to the scutellar shield; the elytral cuticle is usually yellow-brown with dark markings in a zig-zag pattern, but may be mostly plain, without patterning; the depressed frontal area between the eyes has a narrow, inverted Y-shaped sulcus; and abdominal ventrite 3 has a prominent, raised, golden yellow setal patch extending the full length of the ventrite (Fig. 18b).

In contrast, males of *T.felix* (Figs 13, 14) are much larger than those of *T.cekalovici* sp. nov.; the pronotal cuticle is dark brown to black with pale lateral margins and no yellow discal markings; the elytral cuticle is dark brown or red-brown, with setal patterning only; the depression between the eyes does not have an inverted Y-shaped sulcus; and abdominal ventrite 3 has a median, golden yellow setal patch that is not distinctly raised and does not extend the entire length of the ventrite (Fig. 13b).

The aedeagi (Figs 14, 19) of the two species are clearly different. In *T.cekalovici* sp. nov. (Fig. 19) the parameres have curved lateral margins and curved medial margins, and the apices are broad. In *T.felix* (Fig. 14), the parameres have straight lateral margins and only slightly curved medial margins, and the apices are narrow.

**Figures 21–23.**
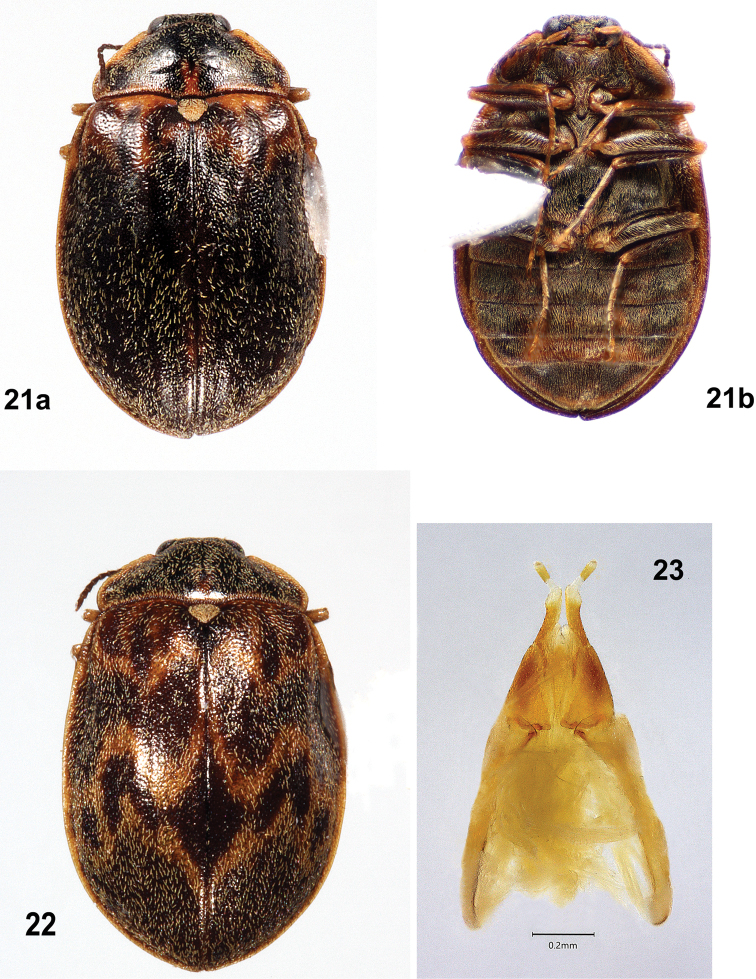
*Tychepsephuscekalovici* sp. nov., female **21** habitus **a** dorsal view **b** ventral view; length 4.4 mm **22** dorsal color pattern variation; length 4.3 mm **23** ovipositor, dorsal view.

Like the males, females of *T.cekalovici* sp. nov. (Figs 21, 22) are much smaller (3.3–3.9 mm long) than females of *T.felix* (4.3–5.7 mm long), and most have elytra with transverse yellow bands in a zig-zag pattern. Individuals of *T.cekalovici* without elytral patterning usually may be distinguished by a mediobasal yellow or yellow-brown spot, sometimes faint, on the pronotum anterior to the scutellar shield. In contrast, females of *T.felix* (Fig. 15) have brown elytra without yellow banding, and do not have a mediobasal yellow spot on the pronotum.

#### Description.

**Male** (Figs 18–20). ***Body***: dorsally and ventrally covered with moderately long, coarse, yellow setae and shorter, thinner, silky, black setae; cuticle with closely spaced punctures, punctures finer ventrally. Cuticle of head, pronotum and venter dark brown to black; pronotum with yellow margins; elytra usually yellow-brown with dark brown patterns. Length 3.3–3.9 mm, width 2.0–2.6 mm (*n* = 19). ***Head***: covered with moderately long, yellow setae. Vertex between eyes slightly wider than diameter of an eye. Frons deflexed at 90° angle from vertex; moderately deep depression between eyes containing a narrow, inverted Y-shaped sulcus, arms of Y deeply incised. Frontoclypeal suture absent. Clypeus wider than long, narrow at base; anterolateral angles curved beneath antennal bases. Maxillary palpus with four palpomeres; palpomeres 1–3 yellow-brown; 4 dark brown, obliquely hatchet-shaped, weakly curved at apex. Labial palpus light to dark brown, with three palpomeres, palpomere 3 truncate to weakly curved; glossae and paraglossae apically bifid and acicular. Antenna weakly serrate, with 11 antennomeres; 1 longest, cylindrical, yellow; 2 half as long as 1, yellow-brown; 3–11 dark brown, each wider apically. Antennal base encircled by raised margin. Eye large, bulbous, finely faceted. ***Pronotum***: a little more than twice as wide as long, widest just anterior to base; all margins yellow; anterior margin slightly sinuate, convex between anterior angles; anterior angles prominent, broadly rounded, projecting anteriorly, clasping eyes; lateral margins finely sculptured with shallow notches, margins straight to basal 1/3 then curved to posterior angles; posterior angles slightly obtuse; posterior margin crenulate, straight laterally then curved to scutellar shield, straight anterior to scutellar shield; disc convex at middle, depressed near anterior angles; disc anterior to scutellar shield with an oblong, subbasal yellow spot and a short, shallow, median sulcus. ***Scutellar shield***: pentagonal, as long as wide; anterior margin crenulate; disc flat, depressed, densely covered with coarse yellow setae. ***Elytra***: conjointly longer than wide, widest ~ 1/3 the distance from apices, wider than pronotum; each elytron with anterior margin crenulate; lateral margin yellow, smooth, narrowly explanate; humerus moderately prominent; elytral base depressed between humerus and scutellar shield; area adjacent to lateral margin in anterior 1/2 with a wide, moderately deep depression; disc more convex in posterior 1/2. Disc densely punctate, punctures small, separated by less than own width; setose, setae less dense near base and along suture. Cuticle variably patterned, usually yellow with transverse, zig zag bands of dark brown spots near the base and at the apical 1/3, sometimes lacking the apical band. Epipleuron widest at humeri, narrowing at abdominal ventrite 1, grooved adjacent to abdomen. ***Metathoracic wings***: macropterous. ***Prosternum***: wider than long; anterior margin projecting to cover mouthparts with a chin piece; prosternal process extending past procoxae, medially with weak longitudinal carina, lateral margins carinate. ***Mesoventrite***: strongly transverse; mesoventral process triangular, extending beyond mesocoxae; mesoventral cavity deep to receive prosternal process. ***Metaventrite***: wider than long, dark brown to black; disc flattened at midline and strongly convex laterally; deep fossa at junction of discrimen and metakatepisternal suture. ***Legs***: similar, except procoxa and mesocoxa globular, metacoxa very short and transverse; femur and tibia usually darker than tarsus; tibia and tarsus long and narrow, tarsus longer than tibia; tarsus usually with dense, short, pale setae; claws short, slender. ***Abdomen***: mottled yellow-brown and dark brown, densely setose; with five ventrites, 1 shortest, 5 longest; ventrites 1–3 very convex in middle; ventrite 3 with prominent, raised, oval, medial patch of dense, coarse, golden yellow setae. ***Aedeagus***: (Fig. 19) 2.5X as long as wide; phallobase narrow basally then widening apically; parameres stout, much longer than penis and enclosing it, apices with inner margins truncate; penis much narrower than a paramere, elongate, parallel-sided, broadly rounded at apex, with broad basal apophyses. **Female** (Figs 21–23) larger than male and darker. Elytra red-brown or dark brown, usually patterned with several yellow, transverse, zig zag bands; less often with yellow markings only at base. Pronotum with a small, triangular, yellow or yellow-brown subbasal spot just anterior to scutellar shield, sometimes obscure. Tarsi not densely setose. Abdominal ventrite 3 lacking a prominent, raised, medial patch of golden yellow setae. ***Ovipositor***: (Fig. 23) with baculus nearly twice as long as gonocoxite; baculus almost twice as long as wide, strap-like, wider apically; each proximal gonocoxite triangular, distal gonocoxites separate basally then converging to meet apically, apices obliquely truncate; each gonostylus long and narrow, half as long as distal gonocoxite. Very similar to that of *T.felix* (Fig. 16).

#### Variation.

Males are smaller than females: males, 3.3–3.9 mm long, 2.0–2.6 mm wide (*n* = 19); females, 4.3–4.7 mm long, 2.1–3.0 mm wide (*n* = 7). The elytral cuticle of males is yellow, yellow-brown, or brown with dark brown patterning (Figs 18, 20), while that of females is red-brown to dark brown with yellow patterning (Figs 21, 22). The pronotal yellow, oblong, subbasal spot of the males is usually yellow-brown and reduced to a triangle or less in the females. The tibiae of males are more setose than those of females. Females (Fig. 21b) lack the prominent, raised patch of golden yellow setae on abdominal ventrite 3 that is present in males (Fig. 18b).

#### Egg description.

Eggs spherical, 0.2 mm diameter (*n* = 10); flattened on one side; chorion with tiny dimples.

#### Etymology.

The trivial name, *cekalovici*, honors the late Tomás Čekalović, who was an outstanding coleopterist, arachnologist, and field biologist from Concepción, Chile (Urbina Burgos 2013). The name is a noun in the genitive case.

#### Geographic distribution.

*Tychepsephuscekalovici* sp. nov. is known only from Chile. Adults have been collected in Región VII (del Maule), Región VIII (Bío Bío) (M. Elgueta, in litt.), Region XIV (Los Ríos), and Región X (Los Lagos), in both the Andean and the coastal mountain areas (Fig. 7). The greatest number of adults collected by the authors was at Río Colegual west of Puerto Varas (Fig. 8).

#### Habitat.

*Tychepsephuscekalovici* sp. nov. adults were found in habitats as described under the genus *Tychepsephus* (see above). Adults were collected by sweeping marginal vegetation along streams and small, shallow rivers during the austral summer (Figs 8, 9, 11).

#### Associated dryopoid taxa.

Elmidae: Larainae: *Hydoraannectans*, *H.lenta*; Elminae: *Austrolimnius*, *Luchoelmis*, *Neoelmississicollis*. Both *T.cekalovici* sp. nov. and *T.felix* occurred at Río Colegual.

### ﻿ Eubrianax luteosignatus

Taxon classificationAnimaliaColeopteraPsephenidae

Pic, 1947

D370D189-23EC-5BFC-BCAA-D12B8CA9810A

Fig. 24

#### Type locality.

“Chili”.

#### Material examined.

**Figure 24.**
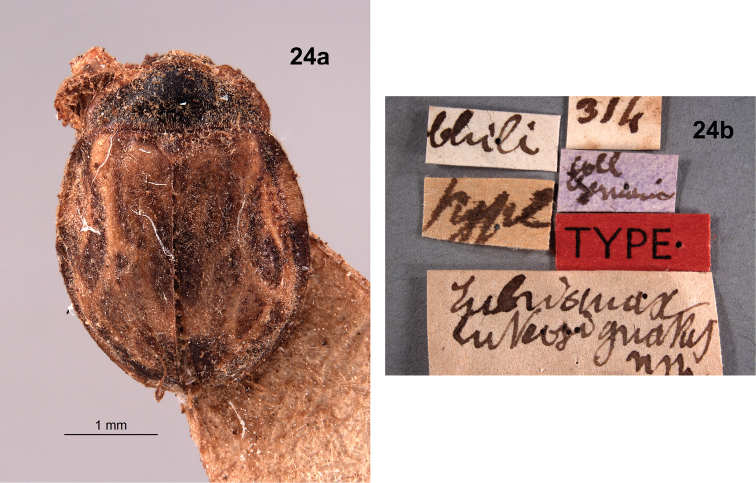
*Eubrianaxluteosignatus*, type specimen **a** dorsal habitus **b** specimen data labels. Images provided by Christophe Rivier (MNHN).

***Holotype*.** Chile: “Chili // type // Eubrianax luteosignatus nsp // 314 // coll Germain [lavender label] // TYPE [red label]” (Fig. 24). Deposited in the MNHN.

#### Remarks.

*Eubrianax* is a Holarctic element that is not expected to occur in Chile. When the type of *Eubrianaxluteosignatus* (Fig. 24) from the MNHN was examined, it was found to be very dirty and glued on a point with the ventral side obscured. We did not seek permission to clean and remount it so our observations were limited. However, it appears that the type actually belongs in the Eubriinae, rather than in the Eubrianacinae where *Eubrianax* is placed. The elytral markings are very reminiscent of the eubriine genus *Dicranopselaphus* which, in the New World, is known from the eastern USA south through Central America. Pic (1947) described *Eubrianaxluteosignatus* in the Dascillidae.

##### ﻿Eubriinae: unknown genus and species of larvae (Chile)

**Figures 25, 26.**
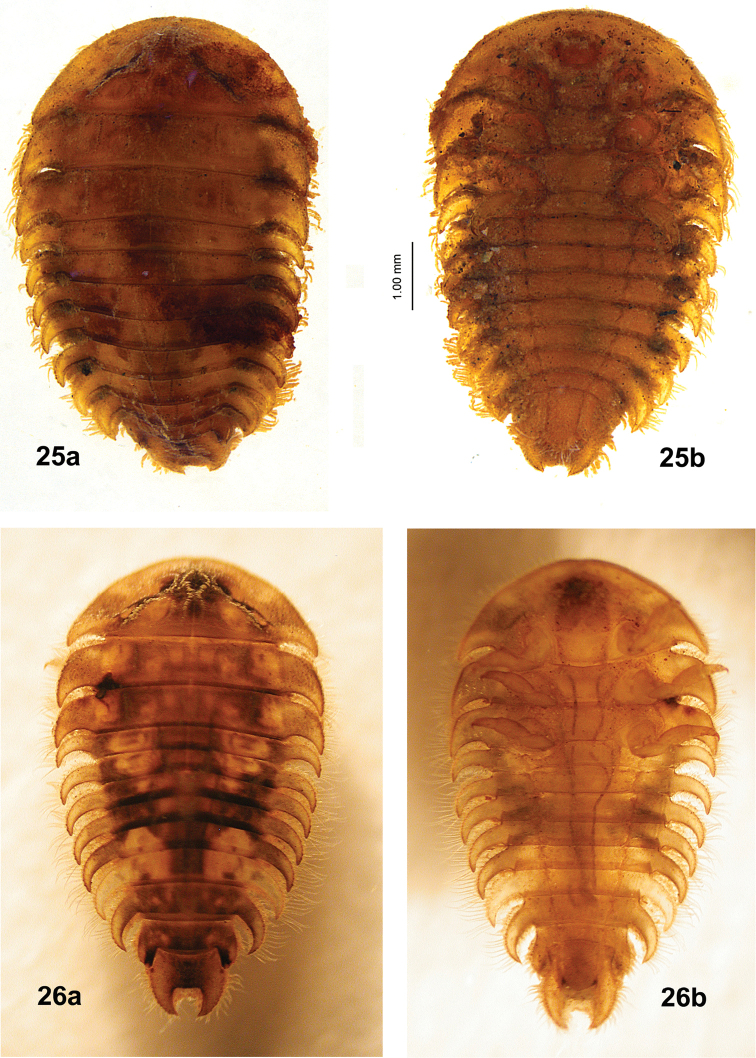
Unknown eubriine larvae from Chile **25** specimen from Estero Nonguén, Concepción **a** dorsal view **b** ventral view **26** specimen from Reserva Nacional Los Ruiles, northwest of Cauquenes **a** dorsal view **b** ventral view. Fig. 26 images provided by Mario Elgueta and Marcelo Guerrero (MNNC).

We know of two larval specimens of an unknown genus and species of Eubriinae from Chile, one in the EMEC and one in the MNNC. The EMEC specimen (Fig. 25) is unfortunately in marginal condition. It was collected by Tomás Čekalović who lived near Concepción, Chile. The locality, Estero Nonguén, flows from Parque Nacional Nonguén through an urban area of Concepción. The other larva (Fig. 26), from the MNNC, is from Reserva Nacional Los Ruiles near the coast northwest of Cauquenas (Elgueta and Guerrero 2005). These two specimens may be the larva of the species currently known as *Eubrianaxluteosignatus*.

##### ﻿Eubriinae: unknown genus and species of larva (French Guiana)

One larva resembling *Tychepsephus* (larval morph 2) (Fig. 6) was collected in a stream in French Guiana (FRENCH GUIANA: Sinnamary, Carbet Mouche, Crique “Salle de Bains”, 04.648440°N, 052.94082°W, elevation 37 m). If this actually is a species of *Tychepsephus*, it would represent a range extension of approximately 4500 km to the northeast of the known distribution of the genus in Chile. Verification would require collection of adult specimens.

## ﻿Discussion

### ﻿Sampling results and larval identification

To verify the taxonomic identity of larvae, one must either collect associated adults, conduct DNA studies, or rear larvae to adulthood. Larvae, if present, are present continuously, but adults are short-lived so the timing of sampling is important if one is to collect adults. Although we visited Chile during the summer when adults are present, our sampling regimen was weighted towards aquatics, and the riparian habitat was not as well-collected. During the 120 collection events, we collected hundreds of larvae in approximately one third of the events (44 times), but adults at only six events at five localities (Fig. 7). The literature and museum records reflect this disparity as well, with mostly larval and few adult records. Timely sampling of the adult habitat would yield more adult specimens, and perhaps additional species of *Tychepsephus* may be discovered. The variability of larval morph 2 points to that possibility.

Larval morph 2 (Fig. 2) was by far the most commonly collected of the two morphotypes. Both morphs co-occurred at only three localities. Of these, Río Colegual (Fig. 8) was the only one where both larval morphs and adults of both species were collected. Adults of both species were collected previously at Corral by another researcher. *Tychepsephuscekalovici* adults were three times more common than those of *T.felix* in our samples.

Association of specific larval morphs of *Tychepsephus* with particular species could be accomplished by either DNA barcoding or by rearing late-instars through to adulthood. Rearing would require holding late-instars (probably collected in November) until they pupate and emerge as adults (likely in December or January). No special equipment would be required. They could be reared in sealable plastic containers that retain humidity, along with some suitable substrate for pupation.

### ﻿Remaining questions

The Chilean psephenid fauna currently includes three, or perhaps four, eubriine species: two species of *Tychepsephus*, *T.felix* (including Ec. (Chilectopria) grandis syn. nov.) and *T.cekalovici* sp. nov., one species currently known as *Eubrianaxluteosignatus*, and one unidentified eubriine larva (Figs 25, 26). Possibly, this unidentified larva is that of *Eu.luteosignatus*. Because Pic sometimes described sexually dimorphic males and females of one species in different genera, it is not surprising that his *Eu.luteosignatus* is probably a eubriine rather than a eubrianacine, and that his Ec. (Chilectopria) grandis is synonymous with *T.felix*. Both *Eubrianax* and *Ectopria* are now considered to be Holarctic genera. Care must be taken when interpreting the literature on *Tychepsephus* because of the uncertainty about which species is being discussed.

*Tychepsephus* has been thought to be endemic to Chile, but older literature records and a recent larval collection from Argentina (Fig. 5) have shown otherwise. Furthermore, an enigmatic larva from French Guiana (Fig. 6), which resembles the larvae of *Tychepsephus*, is particularly important as it potentially represents a very large geographic extension for the genus. Additional specimens, particularly adults, are needed for verification and description of the latter. And sampling in previously uncollected areas would fill in distributional gaps.

The Psephenidae of South America are poorly known and problematic. The monospecific eubriine genus *Neoeubria* occurs in Colombia (W. D. Shepard, unpublished data), Ecuador, and Costa Rica (Shepard and Barr 2014), yet very few adult specimens exist. The more speciose psephenine genera *Pheneps* Darlington, 1936, *Psephenops* Grouvelle, 1898, and *Psephenus* Haldeman, 1853, all have species described from South America. Seven of the nine *Pheneps* species are South American, five of them from Brazil. The Brazilian *Bertrandibicoloripes* Pic, 1943, is actually a species of *Pheneps* (W. D. Shepard, unpublished data). Of the nine valid species of *Psephenops*, only two are known to occur in South America; the remainder are from Mexico, Central America, and the Caribbean. However, the South American species described in *Psephenus* (four from Brazil and Peru) are actually species of *Psephenops*; *Psephenus* occurs only in North and Central America. Additionally, in South America, several undescribed psephenid taxa are known only from larvae. In Colombia, there are undescribed genera and species of Eubriinae and Psepheninae (L. Alvarez, in litt.), in Peru undescribed Psepheninae, and in French Guiana undescribed Eubriinae (W. D. Shepard, unpublished data). South America is a truly fertile territory for further research on the Psephenidae.

## Supplementary Material

XML Treatment for Eubriinae

XML Treatment for Tychepsephus

XML Treatment for Tychepsephus felix

XML Treatment for Tychepsephus cekalovici

XML Treatment for Eubrianax luteosignatus

## ﻿Appendix 1

Chilean localities where *Tychepsephus* was collected by the authors. Sites where adult specimens were collected are marked with an asterisk, *. Region designations are as at the time of the collection.

**Región VIII, Bío Bío** (some of these are now in Región XVI, Ñuble)

Termas de Chillan area (road to ski lift), unnamed stream, 5530 ft, 10 I 2002, 36°55'02"S, 71°25'45"W, larvae (WDS-A-1432)

Termas de Chillan area (across road to Cueva Los Pincheira), Estero Renegado, 4100 ft, 10 I 2002, 36°53'46"S, 71°33'03"W, larvae (WDS-A-1434)

Cueva Las Pincheira, unnamed stream, 4100 ft, 10 I 2002, 36°53'46"S, 71°33'03"W, larvae (WDS-A-1435)

Puente San Jorge, 3 km NW Predio San Jorge, Río Culenco, 1780 ft, 11 I 2002, 37°15'30"S, 72°56'00"W, larvae (WDS-A-1438)

**Región IX, Araucanía**

Parque Nacional Nahuelbuta, picnic area, Estero Pehuenco, 3656 ft, 12 I 2002, 37°49'47"S, 73°00'32"W, larvae (WDS-A-1439)

Parque Nacional Nahuelbuta, picnic area, Estero Pehuenco, 3659 ft, 5 I 2003, 37°49.702'S, 73°00.622'W, larvae (WDS-A-1732)

Parque Nacional Nahuelbuta, Estero Los Gringos, 4167 ft, 12 I 2007, 37°48'21"S, 73°00'45"W, larvae (WDS-A-1440)

Parque Nacional Nahuelbuta, Estero Los Gringos, 4043 ft, 5 I 2007, 37°48.517'S, 73°01.000'W, larva (WDS-A-1733)

14 road km east of P. N. Nahuelbuta, Puente El Manzana, Río Piculquén, 2850 ft, 12 I 2002, 37°48'42"S, 72°52'41"W, larva (WDS-A-1441)

1 road km NE Puente El Manzana, unnamed trib. to Río Piculquén,2960 ft, 12 I 2002, 37°47'51"S, 72°51'20"W, larvae (WDS-A-1442)

19 road km E Victoria, Puente Quino, Río Quino, 2080 ft, 13 I 2002, 38°17'21"S, 72°10'00"W, larvae (WDS-A-1443)

33 road km E Victoria, Puente Huillinlebu, 2720 ft, 13 I 2002, 38°19'05"S, 72°00'49"W, larvae (WDS-A-1444)

42 km E Victoria, Puente Rari Ruca, 2740 ft, 13 I 2002, 38°20'13"S, 71°58'15"W, larvae (WDS-A-1445)

Parque Nacional Huerquehue, Refugio Tinquilco parking area,unnamed stream, 3640 ft, 15 I 2002, 39°09'27"S, 71°42'52"W, larva (WDS-A-1452)

3–4 km S Paillaco, Puente Huepil, Río Liucura, 2440 ft, 15 I 2002, 39°13'57"S, 71°45'20"W, larvae (WDS-A-1454)

**Región X, Los Lagos** (some of these are now in Región XIV, Los Ríos)

4 km SE Coñaripe, Río Llancahue (tributary of Lago Calafquen), ~ 39°34'52"S, 71°57'28"W, 16 I 2002, larva

Parque Nacional Puyehue, 7 km S Aguas Calientes, Río Nauto, 3130 ft, 17 I 2002, 40°45'25"S, 72°17'42"W, larvae (WDS-A-1460)

ca. 3 km SW Ensenada at Highway 225, unnamed stream, 1340 ft, 18 I 2002, 41°14'03"S, 72°36'03"W, larvae (WDS-A-1462)

3 km W Nueva Braunau, 13 km west Puerto Varas, Puente Colegual, Río Colegual, 1200 ft, 18 I 2002, 41°19'34"S, 73°07'20"W, larvae (WDS-A-1463)

*3 km W Nueva Braunau, Río Colegual, 30 XII 2002, 41°19.58'S, 73°07.35"W, adults (WDS-A-1502)

*3 km W Nueva Braunau, Río Colegual, 8 I 2003, 41°19.58'S, 73°07.35"W, adults

8 km W Loncotoro, unnamed stream, 1270 ft, 19 I 2002, 41°18'01"S, 73°18'27"W, larvae (WDS-A-1464)

9 km E Loncotoro, Puente Colegual 2, Río Colegual, 700 ft, 19 I 2002, 41°16'30"S, 73°06'31"W, larvae (WDS-A-1465)

*9 km E Loncotoro, Puente Colegual 2, Río Colegual, 700 ft, 8 I 2003, 41°16.51'S, 73°06.52'W, adults (WDS-A-1519)

Isla Chiloé, 13 road km W Chacao, Río Huicha, 1000 ft, 19 I 2002, 41°52'51"S, 73°39'29"W, larvae (WDS-A-1466)

Isla Chiloé, 5 km N Lago Tarahuín, unnamed stream, 180 ft, 20 I 2002, 42°40'30"S, 73°47'46"W, larva (WDS-A-1467)

Isla Chiloé, 1 road km E Cucao, Puente Curahuelvo, unnamed stream, 50 ft, 21 I 2002, 42°38'31"S, 74°05'38"W, larvae (WDS-A-1469)

Isla Chiloé, Parque Tepuhueico, Río Bravo, 68 ft, 3 III 2008, 42°44.392'S, 73°57.611'W, larvae (WDS-A-1781)

Corral, 2 km SE Aguada, Estero La Aguada, 160 ft, 22 I 2002, 39°54'07"S, 73°25'16"W, larvae (WDS-A-1471)

14 road km SE Corral, Puente Central, Estero Los Llanos, 120 ft, 22 I 2002, 39°57'21"S, 73°22'28"W, larvae (WDS-A-1472)

20 road km SE Corral, Puente Las Romazas, Estero de la Romaza, 60 ft, 22 I 2002, 39°57'41"S, 73°19'33"W, larvae (WDS-A-1473)

*10 km NE Chamiza, Puente Hondo (Río Oroco), 420 ft, 31 XII 2002, 41°28.83'S, 72°48.20'W, adults (WDS-A-1504)

5 km NE Chamiza, Río Chamiza, 360 ft, 31 XII 2002, 41°26.57'S, 72°49.19'W, larvae (WDS-A-1506)

1 km N Contao, Puente Zambo, 440 ft, 1 I 2003, 41°48.30'S, 72°42.72'W, larvae (WDS-A-1507)

*4 km S Contao, Puente Puñon, 850 ft, 1 I 2003, 41°49.84'S, 72°41.95'W, adult elytron (WDS-A-1508)

*6 km E Contaco, Puente Contaco, Río Contaco, 520 ft, 9 I 2003, 40°35.91'S, 73°29.67'W, adult and larvae (WDS-A-1521)

6 km W Contaco, Puente El Avion, 470 ft, 9 I 2003, 40°35.04'S, 73°37.52'W, larvae (WDS-A-1522)

8 km E Puerto Austral, Puente Queche, 640 ft, 1 I 2003, 41°58.58'S, 72°39.72'W, larvae (WDS-A-1510)

*12 km S Caleta Gonzalo, unnamed stream (trib. Río Blanco), 520 ft, 3 I 2003, 42°49.34'S, 72°42.73'W, adult and larvae (WDS-A-1511)

13 km S Caleta Gonzalo, Puente Camahueto No. 1, 600 ft, 3 I 2003, 42°49.55'S, 72°43.44'W, larvae (WDS-A-1512)

3 km S El Amarillo, unnamed tributary of Río Yelcho, 4 I 2003, ~260 ft, 43°02.29'S, 72°28.23'W, larvae

Chaihuin, Reserva Costera Valdiviana, unnamed stream, 686 ft, 12 I 2007, 39°59.884'S, 73°38.901'W, larva (WDS-A-1734)

Chaihuin, Reserva Costera Valdiviana, unnamed stream, 686 ft, 26 II 2008, 39°58.179'S, 73°34.225'W, larvae (WDS-A-1780)

**Región XI, Aysén**

6 km N La Junta, unnamed stream, 590 ft, 4 I 2003, 43°55.34'S, 72°22.66'W, larvae (WDS-A-1515)

3 km S La Junta, unnamed stream, 610 ft, 4 I 2003, 43°59.69'S, 72°24.74'W, larvae (WDS-A-1516)

## ﻿Appendix 2

These *Tychepsephus* locality records are from the literature and museum specimen data, not including that cited in the Material examined sections. Where available, citations include geographic location, date, collector, life stage, and source of information. Region designations are as at the time of collection.

CHILE

**Región Metropolitana**

Chile, Peñaflor, 24 Sep 1896, (larva) (crustacéiforme de Pseudo-Névroptère) (Lataste 1897a, 1897b)

**Región VII, Maule**

Chile, RN Altos del Lircay, Las Majadillas, 6–9 Dic. 2005, Alejandro Vera (*T.cekalovici*) (MNNC)

Chile, Fundo El Rosal, Constitución, 16–30 Oct 1994, G. Arriagada (*T.cekalovici*) (MNNC)

Chile, Parque Nacional Los Ruiles (larva) (Elgueta and Guerrero 2005)

Chile, Tregualemu (larva) (Elgueta and Guerrero 2005)

**Región VIII, Bío Bío**

Chile, Arauco, Laraquete, Río “Los Cruces”, 11 Oct 1958, Fidel Jeldis (larva) (Artigas 1963)

Chile, Cordillera de Chillán, Cueva de los Pincheira, 22 Jul 1962, J. Stuardo y G. Sanhuea (larvae) (Artigas 1963)

Chile, Cordillera Chillán, Pte. Marchant, 15 Ene 1978, Vidal-Taima (*T.cekalovici*) (MNNC)

Chile, Tomé, Río Collén, 21 Abr 1960, André Hulot (larvae) (Artigas 1963)

Chile, Chile, Ñuble, Quirihue, Ene 1988, G. Moreno (*T.felix*) (MNNC)

Chile, Parque Nacional Tolhuaca (larva) (Elgueta and Guerrero 2005)

**Región IX, Araucanía**

Chile, Malleco, PN Conguillío, 20 Dic 1924, F. Ledesma (*Ectopriagrandis*) (MNNC)

**Región XIV, Los Ríos**

Chile, Valdivia, 6 Dic 1980, E. Krahmer (*T.felix*) (MNNC)

Chile, Valdivia, 8 Dic 1980, E. Krahmer (*T.cekalovici*) (MNNC)

Chile, Valdivia, Sto. Domingo, 13 Dic 1981, E. Krahmer (*T.cekalovici*) (MNNC)

Chile, Valdivia, Sto. Domingo, 16 Dic 1984, E. Krahmer (*T.cekalovici*) (MNNC)

Chile, Valdivia, Santo Domingo, 1/15 Dic 1981, E. Krahmer (*T.felix*) (MNNC)

ARGENTINA

Argentina, Provincia del Neuquén, Río Negro basin (larva) (Ectopria (Chilectopria) grandis) (Wais 1990)

Argentina, Provincia del Neuquén, Río Meliquina (larva) (*Chilectopriagrandis*) (Wais 1995)
